# Super‐Enhancer Reprograming Driven by SOX9 and TCF7L2 Represents Transcription‐Targeted Therapeutic Vulnerability for Treating Gallbladder Cancer

**DOI:** 10.1002/advs.202406448

**Published:** 2024-11-04

**Authors:** Siyuan Yan, Zhaonan Liu, Teng Wang, Yi Sui, Xiangsong Wu, Jiayi Shen, Peng Pu, Yang Yang, Sizhong Wu, Shimei Qiu, Ziyi Wang, Xiaoqing Jiang, Feiling Feng, Guoqiang Li, FaTao Liu, Chaoxian Zhao, Ke Liu, Jiayi Feng, Maolan Li, Kwan Man, Chaochen Wang, Yujie Tang, Yingbin Liu

**Affiliations:** ^1^ Department of Biliary‐Pancreatic Surgery Renji Hospital Affiliated to Shanghai Jiao Tong University School of Medicine Shanghai 200120 P. R. China; ^2^ State Key Laboratory of Systems Medicine for Cancer,Shanghai Cancer Institute Renji Hospital Affiliated to Shanghai Jiao Tong University School of Medicine Shanghai 200127 P. R. China; ^3^ Centre of Biomedical Systems and Informatics, ZJU‐UoE Institute Zhejiang University School of Medicine, International Campus Zhejiang University Haining 314400 P. R. China; ^4^ Key Laboratory of Cell Differentiation and Apoptosis of National Ministry of Education Shanghai Key Laboratory of Reproductive Medicine Department of Histoembryology, Genetics and Developmental Biology Shanghai Jiao Tong University School of Medicine Shanghai 200025 P. R. China; ^5^ Department of General Surgery Xinhua Hospital Affiliated to Shanghai Jiao Tong University School of Medicine Shanghai 200092 P. R. China; ^6^ School of Life Sciences and Biotechnology Shanghai Jiao Tong University Shanghai 200240 P. R. China; ^7^ Department of Breast Surgery The Second Affiliated Hospital, Zhejiang University School of Medicine Zhejiang University Hangzhou 310009 P. R. China; ^8^ School of Health Science and Engineering University of Shanghai for Science and Technology Shanghai 200093 P. R. China; ^9^ Department of Biliary Tract Surgery I Shanghai Eastern Hepatobiliary Surgery Hospital Shanghai 200438 P. R. China; ^10^ Department of Surgery School of Clinical Medicine LKS Faculty of Medicine The University of Hong Kong Pokfulam Hong Kong SAR 999077 P. R. China; ^11^ Shanghai Key Laboratory of Systems Regulation and Clinical Translation for Cancer Shanghai 200127 P. R. China; ^12^ Department of General Surgery, Jiading Branch Renji Hospital Affiliated to Shanghai Jiao Tong University School of Medicine Shanghai 201800 P. R. China

**Keywords:** core regulatory circuitry, gallbladder cancer, SOX9, super‐enhancer, TCF7L2, transcription‐targeted CDK7 inhibition therapy

## Abstract

Gallbladder cancer (GBC) is a highly aggressive malignancy lacking clinically available targeted therapeutic agents. Super‐enhancers (SEs) are crucial epigenetic cis‐regulatory elements whose extensive reprogramming drives aberrant transcription in cancers. To study SE in GBC, the genomic distribution of H3K27ac is profiled in multiple GBC tissue and cell line samples to establish the SE landscape and its associated core regulatory circuitry (CRC). The biliary lineage factor SOX9 and Wnt pathway effector TCF7L2, two master transcription factor (TF) candidates identified by CRC analysis, are verified to co‐occupy each other's SE region, forming a mutually autoregulatory loop to drive oncogenic SE reprogramming in a subset of GBC. The SOX9/TCF7L2 double‐high GBC cells are highly dependent on the two TFs and enriched of SE‐associated gene signatures related to stemness, ErbB and Wnt pathways. Patients with more such GBC cells exhibited significantly worse prognosis. Furthermore, SOX9/TCF7L2 double‐high GBC preclinical models are found to be susceptible to SE‐targeted CDK7 inhibition therapy in vitro and in vivo. Together, this study provides novel insights into the epigenetic mechanisms underlying the oncogenesis of a subset of GBCs with poorer prognosis and illustrates promising prognostic stratification and therapeutic strategies for treating those GBC patients in future clinical trials.

## Graphical abstract

This study explores epigenetic mechanisms underlying oncogenesis of gallbladder cancer (GBC), revealing SOX9 and TCF7L2 can form a core regulatory circuitry (CRC) to drive oncogenic SE reprogramming and subsequent transcriptional activation of critical malignant genes. The SOX9/TCF7L2 double‐high GBCs exhibit worse prognosis and are susceptible to CDK7‐targeted transcription inhibition therapy.

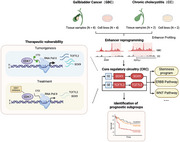

## 1 Introduction

Gallbladder cancer (GBC) is a rare but highly aggressive malignancy with five‐year survival rates of 5%–10%.^[^ ^1^, ^2^ ^]^ Adenocarcinomas (GBAC) constitute the majority of gallbladder cancers, while squamous cell carcinoma (GBSCC) is rare, comprising 1%–4% of cases.^[^ ^3^ ^]^ Surgery and cytotoxic chemotherapy as standard therapies only offer palliation, with almost all patients ultimately succumbing to the disease.^[^ ^4^, ^5^ ^]^ GBC has been extensively characterized at the genomic level by our group and other colleagues, identifying high‐frequency mutations such as *TP53* and *ERBB2* et al.^[^ ^6^, ^7^, ^8^ ^]^ However, its robust genetic heterogeneity presents very limited opportunity for mutation‐targeted therapy.^[^ ^5^, ^7^, ^9^ ^]^ Therefore, alternative approaches are needed for further dissecting the biological basis as well as identifying effective therapeutic strategies against GBC beyond the genetic level.

Epigenetic characteristics, such as histone modifications and chromatin accessibility, portray the cellular cistrome and transcriptome that are remarkably shifted in disease states, including cancer.^[^ ^10^ ^]^ Accumulating evidence has suggested a potential role of epigenetic dysregulation in GBC pathogenesis and therapy.^[^ ^11^, ^12^, ^13^ ^]^ For instance, GBC carries recurrent somatic mutations in genes involved in chromatin remodeling processes, such as *ELF3* (21%), *ARID2* (13%), *ARID1A* (10%), *SMARCA4* (7%), and *HIST1H2AG* (2%).^[^ ^14^ ^]^ Moreover, the histone deacetylase inhibitor PCI‐24781 has been reported to effectively inhibit GBC growth in mice.^[^ ^15^ ^]^ However, our current understanding of the oncogenic function and associated molecular mechanisms of GBC epigenome is mostly confined to non‐coding RNAs^[^ ^16^ ^]^ and DNA methylation at certain gene promoters,^[^ ^12^ ^]^ the cistrome aberration underlying GBC pathogenesis and progression remains elusive.

Enhancers are important epigenetic elements that regulate gene transcription patterns. Active enhancers are marked by H3K27Ac and various transcriptional co‐factors, such as MED1 and BRD4.^[^ ^17^, ^18^ ^]^ The genomic distribution of enhancers in both normal and cancer cells exhibits a similar asymmetric pattern, with a distinct subset (3%–5%) displaying pronounced enrichment in histone acetylation and co‐factor recruitment (up to 40%). These enhancers are commonly referred to as super‐enhancers (SEs), and they control the expression of cell identity genes.^[^ ^19^ ^]^ In cancer, SEs undergo extensive reprogramming and contribute to the aberrant transcriptional activation of oncogenes.^[^ ^20^, ^21^ ^]^ This reprogramming is governed by a few master transcription factors (TFs), which form an interconnected SE‐driven transcriptional network known as the core regulatory circuitry (CRC).^[^ ^22^, ^23^ ^]^ In this feed‐forward network, master TFs not only cooperatively orchestrate SE reprogramming but also serve as SE‐associated target genes themselves.

It has been shown that strong SE‐CRC activity in certain types of cancer leads to “transcriptional addiction” and renders the cancer cells vulnerable to transcription‐targeted therapeutic strategies such as CDK7 inhibition.^[^ ^24^, ^25^, ^26^ ^]^ Transcription inhibition preferentially suppresses the expression of master TFs and SE‐CRC activity, presenting a promising strategy to overcome key challenges of conventional drug therapies, including the lack of druggable targets and the emergence of drug resistance. Notably, multiple CDK7 inhibitor drugs (CT7001 and SY‐1365) have entered early‐phase clinical trials for cancer treatment.

To date, the potential role and the underlying mechanisms of SEs in GBC oncogenesis and therapy remain mostly unexplored. In this study, we aim to comprehensively analyze the SE landscape of GBC and reveal the SE‐CRC regulatory network. Moreover, we investigate the therapeutic potential of SE‐targeted transcription inhibition against GBC in preclinical models.

## 2 Results

### 2.1 Defining the SE Landscape of GBC

To establish the landscape of active regulatory elements in the GBC genome, we employed Cleavage Under Targets and Tagmentation (CUT&Tag) approach to detect the genomic distribution of the active enhancer marker H3K27ac in ten freshly frozen tissue specimens, including eight primary tumor samples of GBC and two benign gallbladder samples of chronic cholecystitis (CC). This selected GBC cohort comprised 7 cases of GBAC and 1 case of GBSCC (Figure S1 and Table S1, Supporting Information). We also performed chromatin immunoprecipitation sequencing (ChIP‐seq) of H3K27ac in four well‐established GBC cell lines (NOZ, GBC‐SD, JXQ‐3D‐4494, JXQ‐3D‐4786). It is noteworthy that the histopathology of all GBC cell lines used in this study is adenocarcinoma.

**Figure 1:**
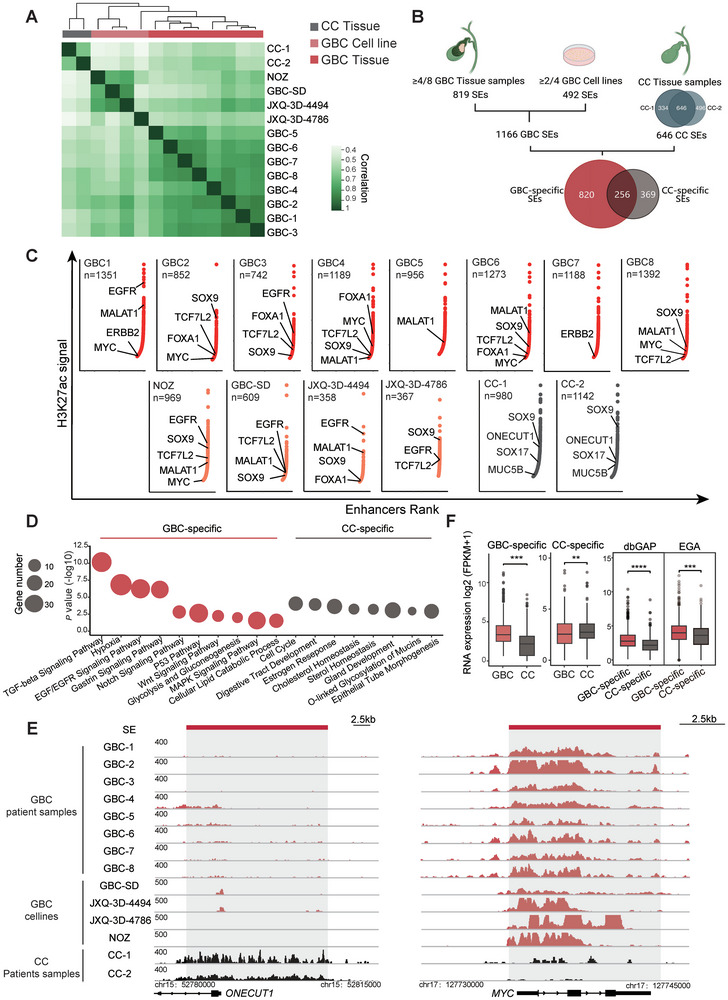
Defining super‐enhancer landscape in gallbladder cancer. A) Hierarchical clustering of the Pearson correlation coefficients among GBC tissue (n = 8), GBC cell lines (n = 4), and CC tissue samples (n = 2) based on H3K27ac signal at SE regions. B) Diagram depicting the strategy for the identification of group‐specific SEs. C) Hockey plot ranking enhancer intensities, and known GBC oncogenes are displayed as examples. D) Functional enrichment of group‐specific SE related genes. E) H3K27ac profiles of the *ONECUT1* and *MYC* loci in GBC and CC samples. F) Box plot of mRNA expression of GBC/CC‐specific SE related genes in corresponding GBC (n = 12) and CC (n = 2) samples (left) and GBC cohorts (EGA, n = 120 and dbGAP, n = 51) (right). Unpaired t test was used for statistical analysis.

Using H3K27ac as a preferred SE identifier, we delineated the genomic distribution of enhancers and SEs among these biological samples. Hierarchical clustering analysis using the total enhancers, or the SE profile of the above‐mentioned samples revealed that only the SE profile could clearly distinguish malignant GBC samples from the control CC samples (**Figure** **1**A,C; Figure S2B, Supporting Information). This suggests that SEs, which experience dramatic reprogramming during GBC tumorigenesis, represent more robust and conserved oncogenic molecular signatures. And the overall distribution of super‐enhancers between GBSCC and GBAC did not exhibit distinctive clustering patterns (Figure S2C, Supporting Information). To dissect the GBC‐specific SE repertoire, we compared the GBC SE profile with that of the benign CC samples and identified 820 GBC‐specific SEs and 369 CC‐specific SEs, respectively (Figure 1B,C; Figure S2D and Table S2, Supporting Information). Functional annotation analysis revealed the GBC‐specific SEs were annotated to multiple well‐known gallbladder malignancy signatures, such as EGF/EGFR (*EGFR, ERBB2*), Wnt (*MYC, TCF7L2*), Notch (*HES1*), TGF‐β (*SMAD3*) and hypoxia (*HIF1A, MALAT1*) pathways,^[^ ^8^, ^27^, ^28^, ^29^, ^30^ ^]^ whereas the CC‐specific SEs were associated normal development and function of gallbladder (*ONECUT1, SOX17, MUC5B*)^[^ ^31^ ^]^ (Figure 1D,E). Moreover, transcriptomic analysis revealed that the genes associated with the GBC‐specific SEs exhibited significantly higher mRNA levels in GBC samples than the ones associated with the CC‐specific SEs (Figure 1F). Together, these data demonstrated GBC carried distinct SE landscape that may play important roles in tumorigenesis via driving transcription of crucial malignancy genes.

### 2.2 Identification of the SE‐Driven Core Regulatory Circuitry in GBC

**Figure 2:**
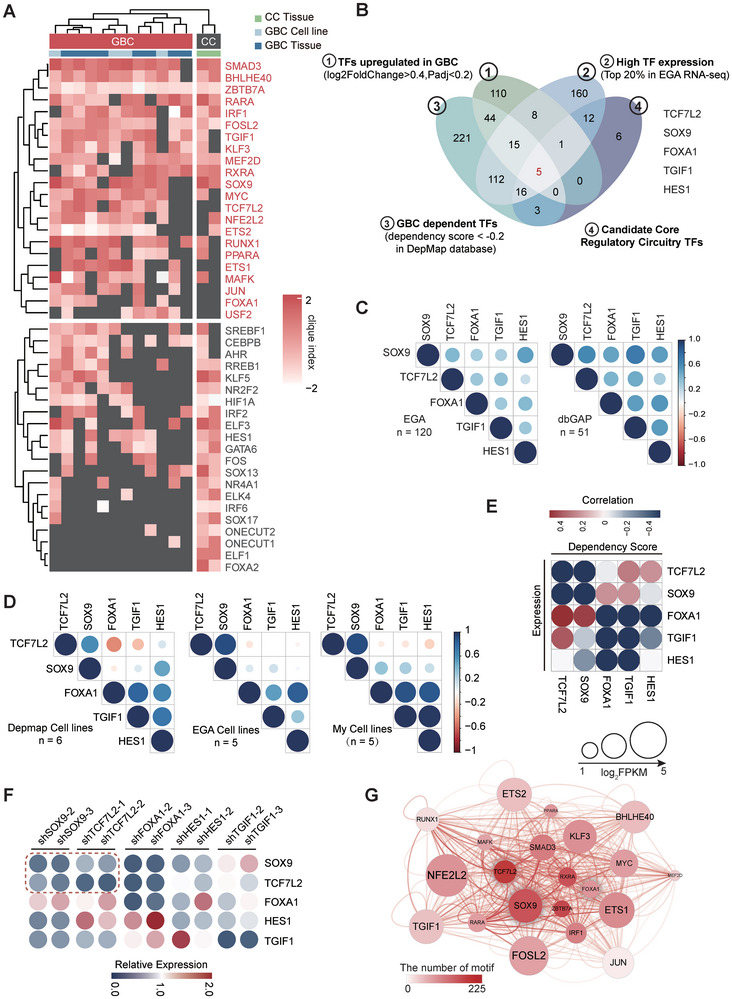
Identification of core regulatory circuitry in gallbladder cancer. A) Heatmap of clique index for the union of CRC TFs across all GBC and CC samples. TFs and samples were clustered by Euclidean distance to reveal the disease status‐specific TF modules. Gray box indicates the TF not associated with any clique in that given sample. The clique enrichment score (percentage of total modules each TF involved in) was normalized by circulating z‐score, termed as clique index. B) Venn diagram showing the integrative analysis for identification of candidate master TFs. C&D) Pearson correlation analysis of master TFs expression in GBC RNAseq cohorts (EGA, n = 120 and dbGAP, n = 51) C), GBC cell lines in DepMap (n = 6), EGA (n = 5), and GBC cell lines available in this study (n = 5) D). The area of circles showed the absolute value of corresponding correlation coefficients. E) Pearson correlation analysis of the dependency score and expression of master TFs among GBC cell lines (n = 6) collected in DepMap database. F) The qPCR results for evaluating the co‐regulation pattern among CRC TFs in NOZ cells with shRNA targeting 5 master TFs. G) Network analysis of CRC TFs in GBC samples, constructed from H3K27ac defined motif‐SE connections. Lines linked TFs whose motifs target each other's SE.

To identify master CRC TFs orchestrating the epigenetic reprogramming in GBC, we utilized the “Coltron” algorithm to analyze the SE profile of GBC and control samples (Figure S2E,F, Supporting Information).^[^ ^20^, ^32^ ^]^ Considering sample heterogeneity, we defined the CRC TFs as those presented in at least 50% of tumor samples’ or in both CC samples’ predictions and identified a total of 43 CRC TF candidates. Unsupervised clustering with normalized clique enrichment score of these CRC TFs could distinguish GBC samples from CC samples, supporting their potential roles in driving SE reprogramming during GBC oncogenesis (**Figure** **2**A; Table S3, Supporting Information). CRC TFs enriched in CC samples included key biliary developmental TFs, such as SOX17 and ONECUT1.^[^ ^31^, ^33^, ^34^ ^]^ CRC TFs enriched in GBC network included TCF7L2, MYC, and FOXA1, known oncogenic TFs overexpressed in GBC.^[^ ^35^ ^]^

Next, we further evaluated the CRC TF candidates of GBC based on the following features as previously defined:^[^ ^23^ ^]^ 1) Significantly upregulated and top expressed in tumor samples, 2) necessity in maintaining tumor malignancy, 3) transcriptional co‐dependency, 4) direct self‐regulation and mutual regulation. With integrative analysis of whole genome CRISPR screening data from DepMap Project, GBC RNAseq data, and the Coltron prediction results, five CRC TFs (*SOX9*, *TCF7L2*, *FOXA1*, *TGIF1* and *HES1*) were found to meet all our defined criteria for gene expression and tumor‐dependency levels (Figure 2B). Pearson's correlation analysis revealed that these five CRC TFs displayed positive correlation with each other in two published GBC RNAseq datasets (Figure 2C) (EGA: EGAS00001003004, dbGAP: phs001404.v1. p1).^[^ ^36^, ^37^ ^]^

When we further investigated the correlation between the expressions of these TFs in three independent cell model datasets, two distinct SE‐CRC TF modules were consistently identified: SOX9‐TCF7L2 and FOXA1‐TGIF1‐HES1 (Figure 2D). Remarkably, the same pattern was consistently observed in the expression‐dependency correlation analysis of GBC cell model datasets from DepMep (Figure 2E; Figure S2G, Supporting Information). When we individually knocked down each of the five CRC TFs in NOZ, a GBC cell line, to measure the effects on the transcription of others, only *SOX9* and *TCF7L2* were found to exhibit mutual transcriptional dependency, as silencing either of them resulted in a significant decrease of both of their mRNA levels (Figure 2F). Intriguingly, upon further reconstructing the interconnected transcription regulatory network in GBC by integrating the Coltron results and GBC RNAseq data, we observed that *SOX9* and *TCF7L2* were the most highly connected GBC TFs, centering the transcription network (Figure 2G). Moreover, the expression levels of *SOX9* and *TCF7L2* are strongly correlated with H3K27ac signal intensity (Figure S3A, Supporting Information), indicating that enhancers may play a dominant role in the regulation of *SOX9* and *TCF7L2* expression. These data collectively indicated that SOX9 and TCF7L2 might constitute a crucial SE‐driven CRC in GBC. Consequently, our subsequent studies primarily focus on these two CRC TFs.

SOX9 is an SRY‐containing homeobox transcription factor^[^ ^38^ ^]^ active in biliary development,^[^ ^39^, ^40^ ^]^ and has been linked to a worse prognosis in biliary tract cancer.^[^ ^41^ ^]^ Likewise, TCF7L2 acts as a co‐effector of the Wnt/β‐catenin pathway involved in biliary development^[^ ^42^, ^43^ ^]^ and carcinogenesis.^[^ ^44^, ^45^, ^46^ ^]^ Among SOX and TCF family members, *SOX9* and *TCF7L2* were the most expressed in GBC (Figure S2H, Supporting Information). Moreover, pan‐cancer expression analysis of the TCGA and CCLE datasets revealed that GBC ranked among the top cancer types in terms of *SOX9* and *TCF7L2* mRNA levels (Figure S4, Supporting Information). To be noted, none of the cancer types and benign CC tissues^[^ ^47^ ^]^ exhibits as strong correlation patterns of SOX9 and TCF7L2 as GBC in all analysis, further underscoring the specificity of SE‐driven SOX9‐TCF7L2 CRC module in GBC (Figure S3B–D, Supporting Information).

### 2.3 SOX9 and TCF7L2 are Indispensable in Maintaining Tumor Malignancy of GBC

**Figure 3:**
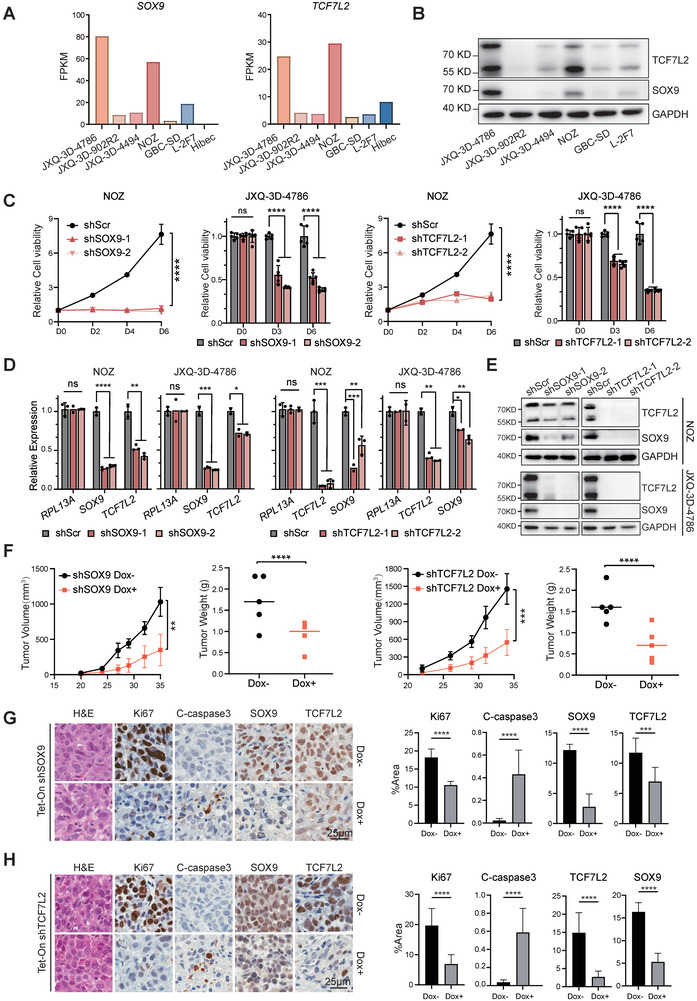
SOX9 and TCF7L2 knockdown impairs tumor growth of GBC. A,B) The mRNA and protein level of SOX9 and TCF7L2 in all available GBC cell lines. C) Cell viability in the NOZ, JXQ‐3D‐4786 cell model following knockdown *SOX9* and *TCF7L2*. Two‐way repeated measures ANOVA was used for statistical analysis with Dunnett multiple hypothesis test correction with five technical replicates. D,E) The qPCR(D) and Western blot (E) results of the coregulation between SOX9 and TCF7L2 in NOZ and JXQ‐3D‐4786 cell line. Unpaired *t* test was used for statistical analysis. F) Xenograft growth curves and tumor weight were shown for Dox‐induced *SOX9* or *TCF7L2* knockdown NOZ cells subcutaneously implanted into athymic mice. For growth curves, two‐way repeated measures ANOVA was used for statistical analysis with Dunnett multiple hypothesis test correction with five technical replicates. For tumor weight, unpaired *t* test was used for statistical analysis. G,H) Representative images and quantification histological analysis. Unpaired *t* test was used for statistical analysis.

To screen for appropriate preclinical models of GBC for further studying SOX9 and TCF7L2, we profiled their expression levels in five available GBC cell lines (JXQ‐3D‐4786, JXQ‐3D‐902R2, JXQ‐3D‐4494, NOZ and GBC‐SD) and two non‐malignant biliary epithelial cell lines (L‐2F7 and Hibec). RNAseq data and immunoblotting revealed that SOX9 and TCF7L2 were highly co‐expressed only in NOZ and JXQ‐3D‐4786, representing the SOX9‐TCF7L2 double‐high GBC models (**Figure** **3**A,B). Upon performing a “loss of function” assay, we revealed that knockdown of either *SOX9* or *TCF7L2* with two individual shRNAs markedly impaired cell viability in both SOX9‐TCF7L2 double‐high GBC lines (Figure 3C). Moreover, knockdown of *SOX9* led to a reduction of *TCF7L2* at transcript and protein levels, and vice versa (Figure 3D,E). RNAseq analysis of NOZ cells upon silencing *SOX9* and *TCF7L2* individually further revealed significant downregulation of themselves and each other's downstream gene signature (Figure S5A,B, Supporting Information), reinforcing the interconnected transcriptional regulation between the two CRC TFs. To be noted, none of the above‐mentioned effects were detected when similar tests were performed on SOX9‐TCF7L2 double‐low cell lines, GBC‐SD and L‐2F7, respectively (Figure S5C,D, Supporting Information). Moreover, exogenous overexpression of *SOX9* and *TCF7L2* in these two lines (Figure S5E, Supporting Information) could neither lead to consistent transcriptional upregulation of the endogenous *SOX9* or *TCF7L2* (Figure S5F, Supporting Information), nor induce cell growth in vitro (Figure S5G–I, Supporting Information).

For the in vivo tests, stable NOZ cell lines with doxycycline‐induced expression of shRNA against either *SOX9* or *TCF7L2* were established, and then were injected subcutaneously into male BALB/c nude mice. Inducible silencing of either *SOX9* or *TCF7L2* potently increased apoptosis and decreased proliferation, resulting in suppression of tumor growth of the SOX9‐TCF7L2 double‐high GBC model in vivo (Figure 3F–H; Figure S5J, Supporting Information). Immunohistochemical staining also confirmed the mutual transcriptional regulation of the two CRC TFs in vivo (Figure 3G,H; Figure S5J, Supporting Information). Taken together, SOX9 and TCF7L2 formed a transcriptional co‐dependent circuit and were indispensable for maintaining tumor malignancy of GBC in vitro and in vivo.

### 2.4 SE‐Associated Transcriptional Autoregulation and Mutual Regulation of SOX9 and TCF7L2 in GBC

**Figure 4:**
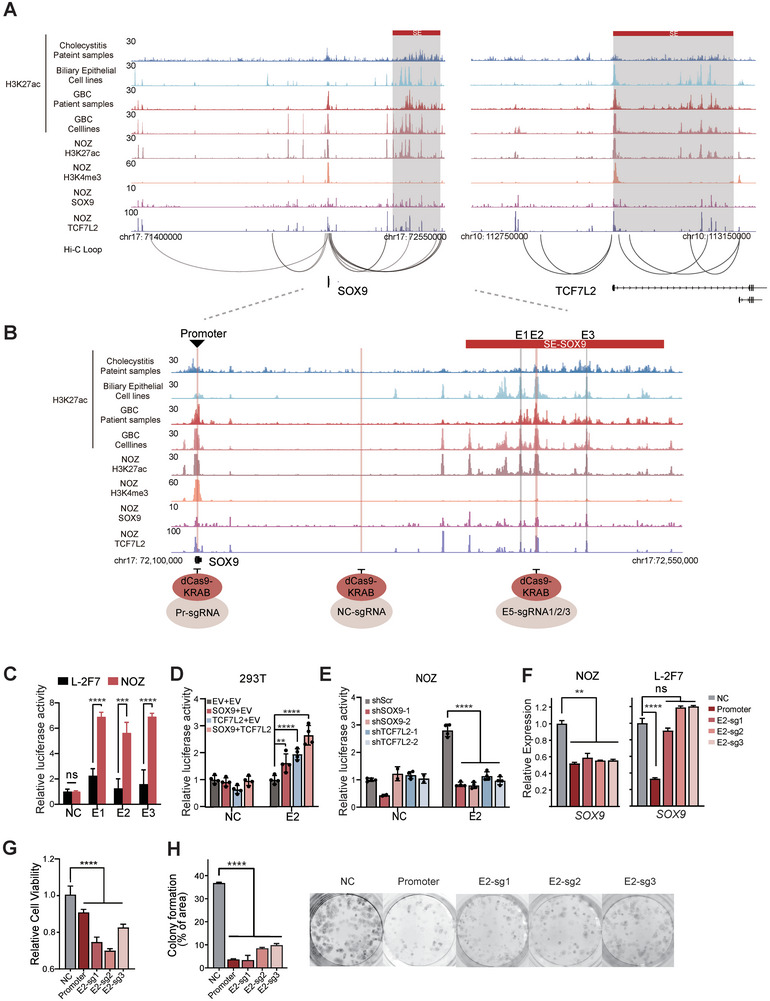
SE‐associated transcriptional coregulation between SOX9 and TCF7L2 in GBC. A) Genome track of CUT&Tag showing co‐localization of SOX9 and TCF7L2 at the promoter and super‐enhancer region of each other. B) Zoom in view of CUT&Tag signals in *SOX9* enhancer locus. C) Enhancer activity assessed by luciferase reporter assays in NOZ and L‐2F7 cells. unpaired *t* test was used for statistical analysis. D&E) Luciferase reporter assay showing the effect of *SOX9* and *TCF7L2* KD/OE on E2 transcriptional activity in HEK293T and NOZ cells. One‐way ANOVA was used for statistical analysis with Dunnett multiple hypothesis test for *p* value correction. F) qPCR results following CRISPRi‐mediated targeting of E2 in NOZ cells. Unpaired *t* test was used for statistical analysis. G,H) CRISPRi‐based cell viability G) and colony formation assay H) in NOZ cells. Unpaired *t* test was used for statistical analysis.

To elucidate the detailed molecular mechanism underlying the interconnected transcriptional regulation between SOX9 and TCF7L2, we employed CUT&Tag to map their genomic occupancy in the SOX9‐TCF7L2 double‐high NOZ cells. As expected, SOX9 and TCF7L2 co‐occupied the promoters and SEs of both two CRC TF genes, forming a mutually autoregulatory loop (**Figure** **4**A). Moreover, analysis of the Hi‐C data of NOZ cells published in our previous study^[^ ^48^ ^]^ identified multiple long‐range chromatin interactions between the promoter and SE regions at the *SOX9* and *TCF7L2* locus, further supporting their direct SE‐driven transcriptional co‐regulation relationship (Figure 4A).

Notably, those identified SEs of *SOX9* and *TCF7L2* could also be detected in some non‐malignant samples, raising the question of whether their roles differ from those in GBC. Given that the results from in situ targeting of *TCF7L2*’s SE would be complicated to interpret due to its location within the gene body region, we next focused on characterizing *SOX9* SE region. We cloned SOX9‐TCF7L2 co‐bound individual constituent enhancer elements into a luciferase reporter vector and observed the robust enhancer activities of E1‐E3 in NOZ cells, but not in the immortalized biliary epithelial cell line L‐2F7 (Figure 4B,C). E2 was then selected for more tests as it exhibited the highest H3K27ac signal in this region. Silencing *SOX9* or *TCF7L2* in NOZ cells reduced the enhancer's activity of E2 (Figure 4D), whereas ectopic expression of *SOX9* and *TCF7L2* in SOX9‐TCF7L2 double‐low 293T cells elevated the E2 activity (Figure 4E). Furthermore, we observed that the co‐overexpression of both CRC TFs led to even stronger activation of the enhancer activity of E2 compared to overexpression of either one alone, providing further evidence for the collaboration of SOX9 and TCF7L2 in activating *SOX9*’s SE. Alternatively, we employed the CRISPR interference (CRISPRi) system, in which single guide RNAs (sgRNAs) guide the dCas9/KRAB complex to restrain targeted cis‐regulatory elements. We designed three independent sgRNA sequences against E2, which markedly decreased the expression of *SOX9* and inhibited GBC cell proliferation in NOZ but not L‐2F7 (Figure 4B,F–H). In contrast, CRISPRi targeting of *SOX9* promoter inhibits its transcription in both lines, which is in line with previous research that the enhancer landscape is more dynamic.

### 2.5 Collaboration of SOX9 and TCF7L2 on Orchestrating SE‐Driven Transcriptional Deregulation in GBC

**Figure 5:**
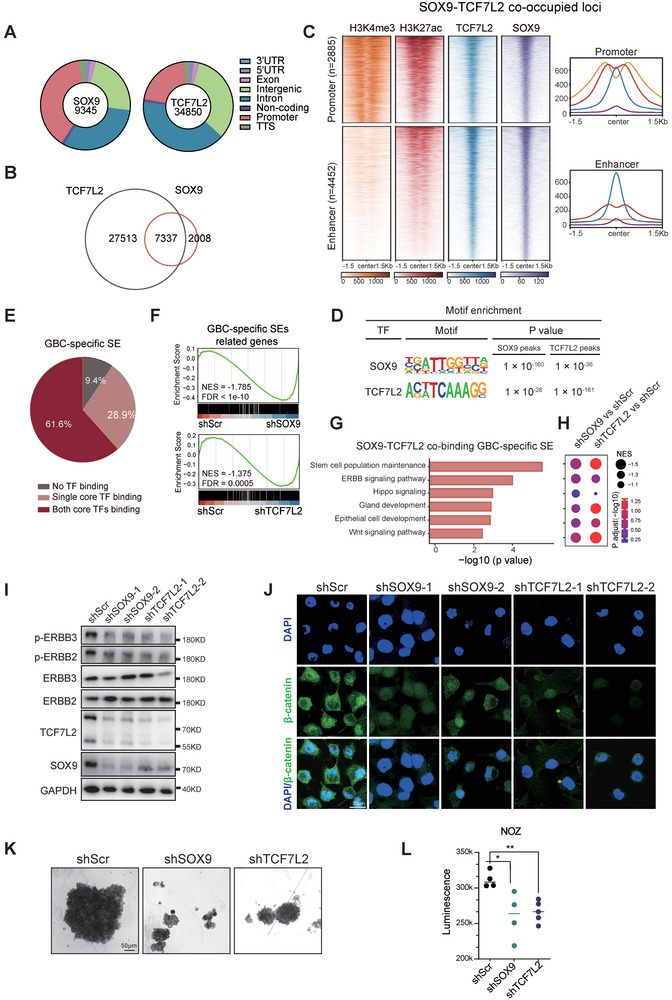
Collaboration of SOX9 and TCF7L2 orchestrates the transcriptional deregulation of GBC. A) Distribution of SOX9 and TCF7L2 peaks in genome. B) The overlap of SOX9 and TCF7L2 binding sites present in NOZ cell line. C) The distribution of CRC TFs, H3K27ac and H3K4me3 signals at SOX9‐TCF7L2 co‐localized region. D) Motif enrichment in SOX9 or TCF7L2 peaks in NOZ cell. E) Proportion of GBC‐specific SE with or without core TFs (SOX9 and TCF7L2) binding in NOZ cell line. F) GSEA results of the GBC‐specific SEs enrichment in NOZ cells with SOX9 or TCF7L2 knocked down. G) Ontology enrichment analysis for the SOX9‐TCF7L2 co‐binding GBC‐specific SEs targeted genes. H) GSEA results of the top enriched gene sets in NOZ cells with SOX9 or TCF7L2 knocked down. I) *SOX9* or *TCF7L2* gene was knocked down in NOZ cells with two independent shRNAs, and the indicated proteins were analyzed by immunoblotting. J) Immunofluorescent images of β‐catenin staining in NOZ cells with *SOX9* or *TCF7L2* knockdown. K,L) *SOX9* or *TCF7L2* gene was knocked down in NOZ cells with shRNAs, and the sphere‐forming capacity was measured by Sphere formation assay (K), and the cell viability was examined by CellTiter‐Glo assays (L). mean ± SD of 3 cell culture replicates and representative phase‐contrast images. Unpaired t test was used for statistical analysis.

We went on to investigate whether the CRC of SOX9 and TCF7L2 drove the transcriptional deregulation of SE‐associated downstream genes in GBC. We identified high‐confidence TCF7L2 (n = 34850) and SOX9 (n = 9345) binding sites in NOZ cells, with the majority located within promoter, intergenic and intron regions (**Figure** **5**A). Upon direct comparison of their cistromes, a substantial genome‐wide overlap of 7337 peaks was observed, consistent with our earlier findings at the gene locus (Figure 5B). Among them, approximately 60.7% (4452 peaks) were situated at enhancer regions, while 39.3% (2885 peaks) were at promoter regions (Figure 5C). Moreover, results from Motif analysis of SOX9 and TCF7L2 not only confirmed the high quality of our CUT&Tag profiling, but also further supported their genomic co‐occupancy (Figure 5D). Furthermore, we were able to detect the interactions between SOX9 and TCF7L2 by co‐immunoprecipitation, which is in line with previously reported interactions between SOX9 and other TCF family proteins in other cancer models (Figure S6A,B, Supporting Information).^[^ ^49^ ^]^

Next, we examined whether SOX9 and TCF7L2 co‐occupied the SE regions to drive the SE reprogramming in GBC. Among the 820 GBC gained SEs identified earlier, 61.6% showed an overlap with both SOX9 and TCF7L2 binding sites, while 28.9% overlapped with either SOX9 or TCF7L2 (Figure 5E). Moreover, the deregulated transcription of GBC‐specific SEs related genes was significantly impaired upon SOX9 or TCF7L2 disruption (Figure 5F), implying the central role of SOX9 and TCF7L2 in the SE reprogramming of GBC. Notably, the SOX9‐TCF7L2 co‐binding GBC‐specific SEs were enriched of multiple GBC‐associated developmental lineage signatures and malignancy gene signatures such as Gland development, Epithelial cell development, Stem cell population maintenance, ErbB signaling pathway and Wnt signaling pathway (Figure 5G).^[^ ^27^, ^29^ ^]^ Knockdown of either *SOX9* or *TCF7L2* resulted in downregulation of transcripts related to these pathways (Figure 5H). Furthermore, we illustrated the key target genes of SOX9‐TCF7L2 driven SE reprogramming within the ErbB pathway (*ERBB2*, *SRC*, *NRP1*), Wnt signaling pathway (*AXIN2*, *FZD2*), and stem cell markers (*BMP4*) (Figure S7, Supporting Information). We also showed that knockdown of *SOX9* or *TCF7L2* in GBC cells resulted in marked suppression of these pathways beyond transcription, including reduced phosphorylation of ERBB (Figure 5I), decreased levels of nuclear β‐catenin (Figure 5J), and a significant impairment of the sphere‐forming capacity of GBC cells (Figure 5K,L).

Collectively, our results illustrated that the CRC formed by SOX9 and TCF7L2 orchestrates transcriptional deregulation of GBC‐associated developmental lineage signatures and malignancy gene signatures through co‐occupying the crucial GBC‐specific SE regions.

### 2.6 Tumor Cell Subpopulation with Higher Levels of SOX9 and TCF7L2 are Enriched of GBC‐Specific SE Gene Signatures and Associated with Worse Prognosis

**Figure 6:**
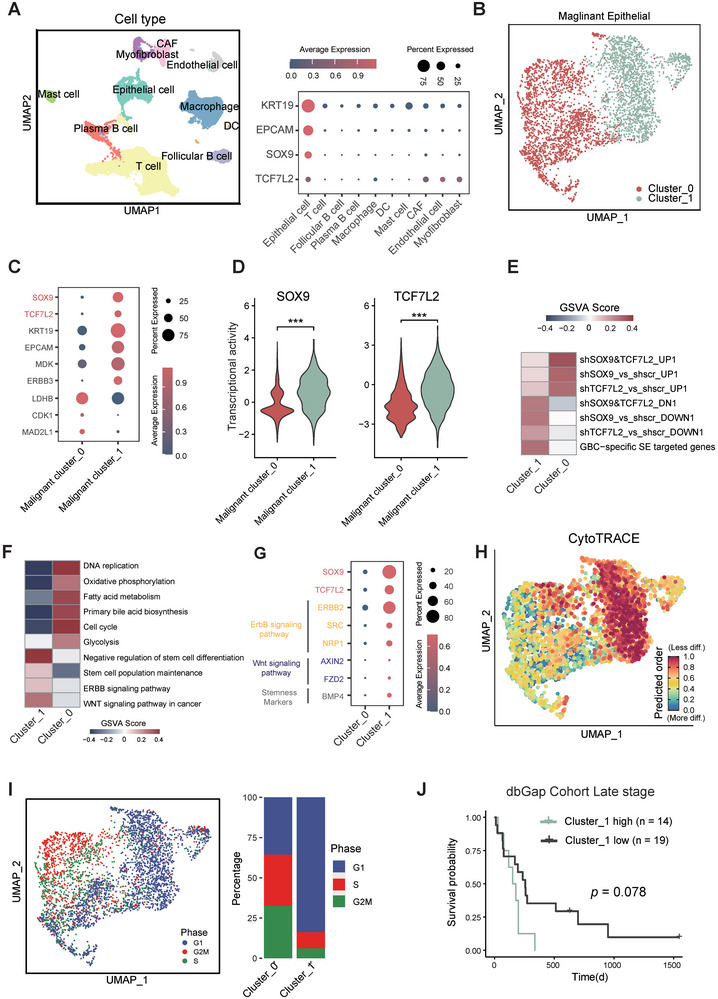
Single cell transcriptome analysis identifies a SOX9‐TCF7L2 double‐high tumor cell subpopulation in GBC. A) UMAP visualization depicting the major cell types identified in scRNAseq data of primary cancer tissues from 9 GBC patients. Bubble plots showing expression levels of CRC TFs and GBC markers among identified cell types. B) Heatmap showing two cell subclusters with differentially enriched gene sets, based on GSVA analysis. C) Bubble plot showing expression of master CRC TFs and malignant gene signatures among epithelial cell subclusters. D) Violin plot showing TF activity of *SOX9* and *TCF7L2* between two cell subclusters. Unpaired t test was used for statistical analysis. E&F) Heatmap showing two cell subclusters with differentially enriched gene sets, based on GSVA analysis. G) Bubble plot showing expression of signature genes in two malignant epithelial cell subclusters. H) The differentiation potential of two malignant epithelial cell subclusters was calculated using the CytoTRACE algorithm. I) Cell cycle analysis was performed to calculate the proportion of cells in different cell cycle phases in two malignant epithelial cell subclusters. J) Kaplan‐Meier curves of overall survival (OS) for an RNAseq cohort of late‐stage gallbladder cancer patients (n = 33) based on the malignant epithelial Cluster_1 proportion deconvoluted with CIBERSORTx. The *p* value was calculated with log‐rank test.

Next, we aimed to further examine the relationship between the CRC of SOX9‐TCF7L2 and its downstream gene signatures in GBC at single‐cell level. We first reprocessed our recently published GBC single‐cell RNAseq data,^[^ ^29^ ^]^ and found “Epithelial cell”, was the only cellular components expressing high levels of both *SOX9* and *TCF7L2* in GBC microenvironment (**Figure** **6**A; Figure S8A,B, Supporting Information). Then, we performed inferCNV analysis to extract the malignant tumor cells of GBC from the “Epithelial cell” subpopulation (Figure S8C, Supporting Information). Unsupervised clustering of those malignant tumor cells identified two major subsets which we named as “Cluster_0” and “Cluster_1” (Figure 6B, Figure S8D–F, Supporting Information). Intriguingly, we found that “Cluster_1” exhibited higher expression level and transcriptional activities of *SOX9* and *TCF7L2* than “Cluster_0” (Figure 6C,D). Consistently, “Cluster_1” was more enriched of the above‐mentioned SE‐associated target gene signatures of the two CRC TFs including Stem cell population maintenance, ErbB signaling pathway and Wnt signaling pathway. In contrast, “Cluster_0” was more enriched of DNA replication and cell metabolism related gene signatures and expressed higher levels of relevant genes such as *LDHB*, *CDK1* and *MAD2L1* (Figure 6E–G). When the two tumor cell subpopulations were further subjected to cell cycle analysis or developmental potential analysis, “Cluster_1” was shown to be much less proliferative and differentiated than “Cluster_0”, which was in line with the above‐mentioned GSVA results (Figure 6H,I). To verify the cell cycle arrest at G1 phase in SOX9/TCF7L2 double‐high cells identified by single‐cell transcriptomic analysis, we utilized multiplex immunohistochemistry (mIHC) to evaluate the protein level of P21, a well‐established marker of G1/S cell cycle arrest,^[^ ^50^ ^]^ in a representative gallbladder cancer tissue sample (Figure S9A, Supporting Information). Our results revealed that SOX9‐TCF7L2 double‐high cancer cells exhibited significantly higher level of P21 compared to SOX9‐TCF7L2 double‐low cells (Figure S9B–D, Supporting Information), supporting a P21‐mediated G1/S cell cycle arrest in SOX9/TCF7L2 double‐high GBC cells.

To be noted, recently published GBC prognostic marker genes, such as *KRT19*, *EPCAM*, *MDK* and *ERBB3*,^[^ ^51^ ^]^ were all found to exhibit higher expression levels in “Cluster_1” (Figure 6C), making us wonder if this tumor cell subpopulation would confer to more severe tumor malignancy and worse prognosis. After performing deconvolution analysis of the transcriptome data of a previously published late‐stage GBC cohort (phs001404.v1. p1 from dbGap), we found that GBC patients with higher percentage of “Cluster_1” tend to exhibit worse prognosis (“Cluster_1 high” group, n = 8; “Cluster_1 low” group, n = 17, *p* = 0.078) (Figure 6J).

Together, our single‐cell transcriptomic analysis demonstrated that the SOX9‐TCF7L2 double‐high tumor cells exhibited GBC‐specific SE gene signatures and was associated with a worse prognosis in GBC.

### 2.7 The Expression of SOX9 and TCF7L2 were Linked to Prognosis in GBC

**Figure 7:**
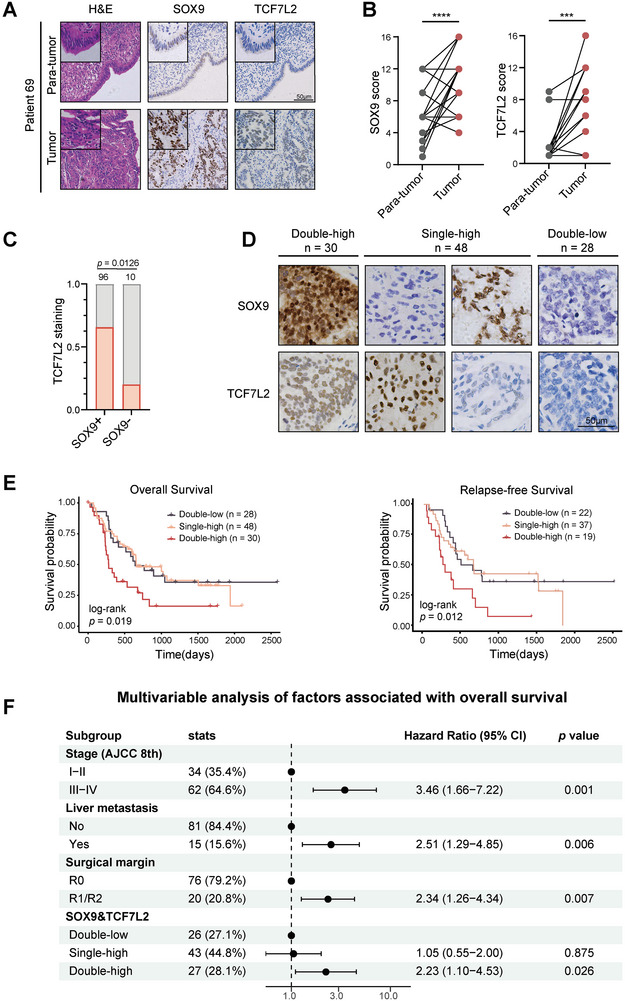
SOX9 and TCF7L2 expression predicts prognosis of GBC patients. A) Representative H&E and immunohistochemical staining of SOX9 and TCF7L2 on paraffin‐embedded tumor and para‐tumor sections of GBC patient 69. Scale bar = 50 µm. B) Quantification of SOX9 and TCF7L2 staining in paired tumor and para‐tumor tissues. C) Quantification of TCF7L2 staining in SOX9 positive (n = 96) or negative (n = 10) GBC tissues (TCF7L2 positive indicated with red bar). D) Representative immunohistochemical staining image of SOX9‐TCF7L2 double‐high, single‐high, or double‐low tumor sections in our GBC tissue cohort (n = 106), respectively. E) Kaplan‐Meier curves of overall survival (OS) and relapse‐free survival (RFS) for GBC patients based on SOX9 and TCF7L2 expression. The *p* value was calculated with log‐rank test. F) Multivariable Cox regression models to evaluate the association between clinicopathological factors and overall survival in the GBC cohort (n = 96).

To further validate the potential prognostic role of SOX9 and TCF7L2 inferred from single‐cell transcriptomic analysis, we profiled their expression in an expanded GBC clinical cohort through IHC (n = 106). In GBC clinical specimens, both TFs were significantly upregulated in tumor compared to paired para‐tumor tissue (**Figure** **7**A,B), and demonstrated analogous correlation (Figure 7C). Of the cases examined, 30 (28.3%) showed nuclear expression of both SOX9 and TCF7L2 (double‐high), 48 (44.4%) expressed only SOX9 or TCF7L2 (single‐high), and 28 (26.4%) cases were double‐low (Figure 7D). Although no significant association between SOX9 and TCF7L2 expression and other clinicopathological factors were shown (Figure S10, Supporting Information), patients with higher levels of SOX9 and TCF7L2 expression exhibited worse overall survival (OS, *p* = 0.019, n = 106) and relapse‐free survival (RFS, *p* = 0.012, n = 78) (Figure 7E). In univariable analysis, we identified significant association between several clinicopathological factors and worse OS (Figure S11, Supporting Information). Among these factors, tumor stage, liver metastasis, surgical margin and SOX9&TCF7L2 staining (double‐high versus double‐low subgroup, HR = 2.23, 95% CI 1.10‐4.53, *p* = 0.026) remained significantly associated with OS in the multivariable analysis (Figure 7F). Hence, the expression levels of CRC components maintained their independent statistical significance even after adjusting for other clinicopathological factors by multivariable analysis. In conclusion, these findings indicated that the expression of SOX9 and TCF7L2 can delineate clinically meaningful tumor subgroups.

### 2.8 SOX9‐TCF7L2 Double‐High GBC Models are Susceptible to SE‐Targeted CDK7 Inhibition Therapy In Vitro and In Vivo

It has been well recognized that SE‐driven transcriptional dependencies can be effectively targeted by CDK7 inhibition therapy in various cancer types.^[^ ^52^ ^]^ Therefore, we tested if CDK7 inhibition therapy could also work effectively on treating SOX9‐TCF7L2 double‐high GBC. As shown in Figure S12A (Supporting Information), results of whole‐genome CRISPR screening from the DepMap project demonstrated strong tumor dependency of CDK7 in all tested GBC lines disregarding the expression levels of SOX9 and TCF7L2. We also performed a parallel in vitro and in vivo CRISPR screening with customed epigenetic‐targeted sgRNA library (6913 sgRNAs targeting 1113 epigenetic‐related genes) in distinct GBC models: the double‐high NOZ model and the double‐low GBC‐SD model (Figure S12B, Supporting Information). The results independently and consistently affirmed the oncogenic reliance on CDK7 in both GBC models in vitro and in vivo, thereby further substantiating its potential as a therapeutic target for GBC (Figure S12C, Supporting Information).

**Figure 8:**
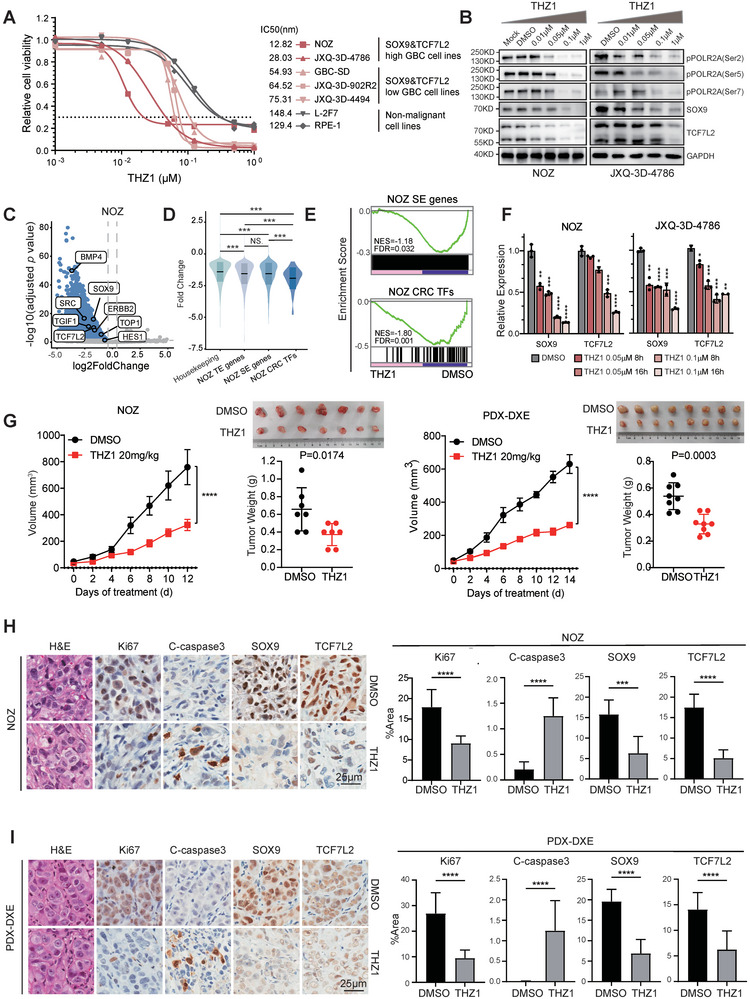
SE‐targeted CDK7 inhibitor THZ1 suppresses SOX9‐TCF7L2 double‐high GBC. A) Cell viability assay testing THZ1 in GBC and non‐malignant cell lines. B) Immunoblot analysis of NOZ or JXQ‐3D‐4786 cells treated with indicated concentrations of THZ1 or DMSO for 24 h. C) Volcano plots showing differentially expressed genes in THZ1 treated NOZ cells (versus DMSO treatment). Genes with adjusted *p* value < 0.05 and log_2_FC < −0.4 are highlighted. D) Changes in gene expression upon CDK7 inhibition with THZ1 for 8 h in NOZ cells. Violin plots are overlaid with box plots of median and quartiles. One‐way ANOVA was used for statistical analysis with Bonferrioni multiple hypothesis test for *p* value correction. E) GSEA results of SE and CRC TFs enrichment in THZ1 treated NOZ cells versus DMSO treatment. F) The qPCR results of *SOX9* and *TCF7L2* expression in NOZ and JXQ‐3D‐4786 cells treated with DMSO or THZ1 for 8 or 16 h. One‐way ANOVA was used for statistical analysis with Dunnett multiple hypothesis test for *p* value correction. G) Tumor proliferation and weight in mice engrafted with NOZ cells or PDX model and treated with DMSO or THZ1. H,I) Representative images (left) and quantification (right) from histological analysis Unpaired *t* test was used for statistical analysis.

Next, we measured the dosage response of the CDK7 inhibitor THZ1 across our panel of the five GBC cell lines (JXQ‐3D‐4786, JXQ‐3D‐902R2, JXQ‐3D‐4494, NOZ and GBC‐SD), with L‐2F7 and the retinal pigment epithelial cell line RPE‐1 serving as non‐malignant controls. Notably, all examined GBC lines demonstrated heightened sensitivity to THZ1 compared to the non‐malignant controls, suggesting a potential therapeutic window in CDK7 inhibition therapy for GBC (**Figure** **8**A). Intriguingly, we noticed that the SOX9‐TCF7L2 double‐high GBC lines (NOZ and JXQ‐3D‐4786) displayed considerably lower IC50 values compared to the others. The on‐target activity of the CDK7 inhibitor was confirmed by the dose‐dependent reduced phosphorylation of the C‐terminal domain (CTD) of RNA polymerase II (POLR2A) in SOX9‐TCF7L2 double‐high GBC cell line upon treatment with THZ1 (Figure 8B). This observation hints that the SE‐driven CRC orchestrated by SOX9‐TCF7L2 within these GBC lines might confer to their heightened sensitivity to SE‐targeted transcription inhibition interventions.

To better understand the underlying molecular mechanisms, we performed RNAseq analysis of THZ1‐treated NOZ cells (0.1 µM, 8 h) and utilized “spike‐in” RNA standards for proper data normalization as previously reported. As depicted in Figure 8C–E, although THZ1 treatment induced a global transcriptional shutdown in NOZ cells, the inhibitory effects were notably more pronounced for SE‐associated target genes and the CRC TF genes than for housekeeping genes, which supports the preferential suppression of SEs through CDK7 inhibition therapy. These findings were validated using qRT‐PCR and western blot (Figure 8B,F). GSEA results also revealed the suppression of multiple SOX9‐TCF7L2 associated downstream malignancy signatures by CDK7 inhibition (Figure S12D, Supporting Information).

The in vivo therapeutic efficacy of THZ1 against GBC was further verified by using two subcutaneous GBC xenograft models. Consistent with the in vitro data, tumor burden in immune‐deficient mice xenografted with NOZ cells was remarkably reduced upon THZ1 treatment, whereas the body weight did not show any significant changes compared with that in vehicle control group (Figure 8G; Figure S12E, Supporting Information). Moreover, IHC staining identified reduced proliferation, increased apoptosis and downregulation of SOX9 and TCF7L2 in THZ1 treated group of mice (Figure 8H; Figure S12F, Supporting Information). Similar results were obtained when conducting above‐mentioned in vivo THZ1 assessment on PDX‐DXE, our self‐established patient‐derived GBC xenograft model also characterized by elevated levels of SOX9 and TCF7L2 (Figure 8G,I; Figure S12F, Supporting Information).

To further clarify the therapeutic potential of CDK7 in treating GBC, we evaluated the effects of another two selective CDK7 inhibitors, SY‐1365 and CT7001, both of which have entered human clinical trials for cancer treatment. As shown in Figure S13 (Supporting Information), SY‐1365 and CT7001, like THZ1, effectively suppressed SE‐driven transcription of SOX9 and TCF7L2 in GBC and exhibited heightened sensitivity against the SOX9‐TCF7L2 double‐high GBC cells.

Taken together, these results demonstrated that the SOX9‐TCF7L2 double‐high GBC models are susceptible to SE‐targeted CDK7 inhibition in vitro and in vivo, thus unveiling a novel and effective therapeutic strategy for those more severe GBCs.

## 3 Discussion

In this study, our epigenetic profiling and characterization reveal a central role of SOX9‐TCF7L2 core transcriptional circuitry (CRC) in the extensive super‐enhancer (SE) associated epigenomic reprogramming in gallbladder cancer (GBC). More importantly, we further illustrate the potential clinical value of the SOX9‐TCF7L2 CRC in GBC. “SOX9/TCF7L2 double‐high” labels not only a subcluster of GBC cells with stem cell‐like features in single‐cell transcriptome analysis, but also a proportion of GBC patients exhibiting earlier recurrence and worse survival. Moreover, “SOX9/TCF7L2 double‐high” GBC models are found to display stronger SE‐driven transcriptional addiction and increased sensitivity to SE‐targeted transcription inhibition therapy. Our study underscores the value of epigenetic profiling and characterization in identifying druggable vulnerabilities of GBC that largely do not manifest as somatic mutations in tumor genome.

SOX9 and TCF7L2, which are known to be active in many developmental and tumorigenesis processes, belong to the SOX and TCF families of TFs, respectively. The interplay between members of the two TF families has been reported before.^[^ ^53^ ^]^ For example, SOX3, SOX4, and SOX17 directly bind to TCFs and β‐catenin and upregulate the expression of downstream target genes.^[^ ^38^ ^]^ As to SOX9, it has long been recognized as a Wnt antagonist that facilitates β‐catenin degradation historically.^[^ ^54^, ^55^, ^56^, ^57^ ^]^ However, recent reports reveal it is required for TCF7‐induced tumor cell proliferation in cholangiocarcinoma^[^ ^58^ ^]^ and correlated with poor prognosis.^[^ ^41^ ^]^ The physical interaction between SOX9 and other TCF family proteins has also been reported in other cancer models before.^[^ ^48^ ^]^ In this study, our findings argue that SOX9 and TCF7L2 could form a feed‐forward loop to cooperatively orchestrate the SE reprogramming in GBC. As SOX9 and WNT signaling have both been implicated in driving normal development of gallbladder and bile ducts, the interplay between SOX9 and TCF7L2 might indicate a SE‐driven oncofetal reprogramming in GBC oncogenesis. Moreover, the co‐expression and co‐dependency relationships between SOX9 and TCF7L2 were more pronounced in GBC compared to other gastrointestinal tumors, suggesting a unique interplay between these two TFs in GBC. Notably, both of our SOX9 and TCF7L2 CUT&Tag data in NOZ cells show significant enrichment of each other's motifs in their binding peak regions, respectively (Figure 5D). Therefore, even though we detected the interaction between SOX9 and TCF7L2 in GBC cells, their co‐localization on chromatin should be largely mediated through their binding to their own respective motifs within the co‐binding peak regions, without necessarily relying on their direct interaction. Further analysis of identifying the co‐binding peak regions containing only SOX9 motifs or TCF7L2 motifs will help better illustrate the potential contribution of direct SOX9‐TCF7L2 interaction to the oncogenic transcriptional reprogramming of GBC.

Although part of the SE regions of *SOX9* and *TCF7L2* in GBC were also enriched of H3K27ac signal and identified as SEs in some non‐malignant samples including the L‐2F7 cell line, our enhancer intervention experiments (Figure 5C–F) revealed that the SE near the *SOX9* locus did not exhibit the similar regulatory activity over *SOX9* transcription in L‐2F7 as it does in NOZ cells. Since the *TCF7L2* SE is unsuitable for enhancer intervention test due to its location within the gene body, we performed an additional SOX9 CUT&TAG analysis in L‐2F7 cells and found no SOX9 binding in the *TCF7L2* SE region (datanot shown). Moreover, no mutual regulatory relationship between SOX9 and TCF7L2 was detected in L‐2F7 (Figure S5D, Supporting Information). These results demonstrated that the SEs identified near the *SOX9* locus or within the *TCF7L2* locus in L‐2F7 cells do not exert similar roles as the SEs of *SOX9* and *TCF7L2* do to form the SE‐associated transcriptional autoregulation and mutual regulation of *SOX9* and *TCF7L2* in GBC cells. More detailed cell type dependent regulatory mechanisms underlying these SE differences required further investigations.

Even though our analysis reveals that enhancer plays a major role in driving activation of SOX9 and TCF7L2 via deregulated transcription, other mechanisms could still exist in GBC. For example, besides the stemness signatures, the ErbB and Wnt pathways are also found to be enriched downstream gene signatures of the SE‐driven SOX9‐TCF7L2 CRC. Intriguingly, SOX9 has been reported to be activated by the ErbB pathway in glioblastoma before.^[^ ^59^ ^]^ As both ErbB and Wnt pathways have been implicated in GBC oncogenesis,^[^ ^8^ ^]^ it would be interesting to further study the potential positive feedback loop involving the two signaling pathways and the SOX9‐TCF7L2 circuitry, which may uncover additional upstream regulatory mechanisms and therapeutic targets. On the other hand, the 17q24.3 region, encompassing the *SOX9* gene, has been reported to be genetically amplified in patients with GBC.^[^ ^60^ ^]^ Moreover, the *SOX9* locus was also found to be significantly amplified in multiple GBC cell lines according to the genomic profiling data from the DepMap database (data not shown).^[^ ^61^ ^]^ These findings are in line with the notion that the prominent driver gene expression can be altered in cancer cells via multiple mechanisms. More importantly, to further illustrate the relationships between the various different deregulatory mechanisms in GBC, such as cooccurrence or mutually exclusive, would better guide development of effective targeted therapy.

Given the overall grim prognosis of GBC, there is an urgent need to identify biomarkers to guide prognosis and clinical management. Our single‐cell transcriptomic analysis and subsequent IHC validation in two independent GBC cohorts proposed SOX9 and TCF7L2 as novel molecular biomarkers for prognostic stratification in GBC. More importantly, we found that GBC preclinical models with higher SOX9‐TCF7L2 expression were more sensitive to SE‐targeted CDK7 inhibition, providing a novel therapeutic strategy for GBC patients with more severe outcome. CDK7‐based transcriptional targeting strategies have shown promising therapeutic effects in preclinical studies, particularly in refractory tumor types lacking conventional drug targets.^[^ ^62^, ^63^ ^]^ Moreover, several CDK7 inhibitors have progressed to clinical trials (NCT03134638, NCT04247126, NCT03770494, and NCT03363893). Hence, our findings lay a solid foundation for future clinical trials assessing CDK7‐targeted therapy for treating SOX9‐TCF7L2 double‐high GBC patients and drive the ongoing exploration of other effective transcription‐targeted small‐molecule inhibitors in this context.

In conclusion, our study provides novel insights into the epigenetic mechanisms underlying GBC oncogenesis and offers promising diagnostic and therapeutic strategies for treating GBC patients in future clinical trials.

## 4 Experimental Section

### Patients and tissue samples

Eight primary gallbladder cancer (GBC) and two chronic cholecystitis samples were surgically obtained from patients and recruited for the study, comprising 7 GBAC and 1 GBSCC. Clinicopathological features of the patients were summarized in Table S1 (Supporting Information). GBC‐1 diagnosis was confirmed via H&E staining. The findings corroborate the single‐cell transcriptome characteristics previously reported for squamous cell carcinoma by the lab. The SCC marker genes (TP63, KRT5) exhibited specific high expression in GBC‐1 was observed (Figure S2A, Supporting Information). The IHC tissue cohort consisted of 106 GBC patients who underwent surgery between August 2014 and December 2021, with a median follow‐up of 539 days (range, 27–2587 days). Variables collected included gender, age, TNM stage (AJCC 8th), surgical margin, et al. All patients’ diagnoses were pathologically confirmed.

### Cell Lines

The 293T and GBC‐SD cell lines were purchased from the Cell Bank of the Chinese Academy of Science (Shanghai, China), and NOZ cells were obtained from the Health Science Research Resource Bank (Osaka, Japan). The GBC primary cell lines (JXQ‐3D‐902R2, JXQ‐3D‐4494, and JXQ‐3D‐4786) were kindly provided by Prof. Jiang Xiaoqing, Shanghai Eastern Hepatobiliary Surgery Hospital (Shanghai, China) and have been previously described.^[^ ^64^, ^65^ ^]^ The immortalized biliary epithelial cell line L‐2F7 was generated and cultured as previously reported.^[^ ^66^ ^]^ NOZ, GBC‐SD, and 293T cells were cultured in DMEM (L110KJ, Basal media) supplemented with 10% FBS (900‐108, Gemini) and 1% penicillin/streptomycin (Gibco). The GBC primary cell lines were maintained in DMEM/F12 medium (Thermo Fisher Scientific) containing 15% FBS (F2442, Sigma), 1% penicillin/streptomycin (Gibco), and 1% MEM Non‐Essential Amino Acids Solution (Gibco). Cell lines were used in experiments within 15 passages from thawing and cultured in a humidified incubator at 37 °C containing 5% CO_2_. The histopathology of all GBC cell lines used in this study was adenocarcinoma.

### Multi‐Omics Data Analysis

The epigenetic and transcriptomic profiling was performed. Sample annotations were provided in Table S1 (Supporting Information). The detailed methods were presented in Supporting Information.

## Conflict of Interest

The authors declare no conflict of interest.

## Author Contributions

S.Y., Z.L., T.W., and Y.S. contributed equally to this work. S.Y.Y. conceived the study, performed the experiments, and interpreted the data. S.Y.Y. and Z.N.L conducted the bioinformatic analysis, visualized the work, and wrote the manuscript. Y.S. technical supported the study. Y.B.L. and X.S.W. performed the radical cholecystectomy. Y.Y. and K.L. collected the patient samples. S.M.Q. conducted the HE staining and pathological diagnosis. T.W. and J.Y.S analyzed the CUT&Tag data and performed the Coltron algorithm. P.P. analyzed the Hi‐C data. C.X.Z. analyzed the single‐cell RNAseq data. S.Z.W. analyzed the bulk RNAseq data with TCGA pipeline. Z.Y.W., F.L.F. and X.Q.J. provided the cell lines. G.Q.L, J.Y.F. and F.T.L. providing resources. M.L.L and K.M. reviewed the manuscripts. Y.B.L., Y.J.T. and C.C.W. supervised the study.

## Supporting information



Supporting Information

Supplemental Table 1

Supplemental Table 2

Supplemental Table 3

Supplemental Table 4

## Data Availability

The data that support the findings of this study are available on request from the corresponding author. The data are not publicly available due to privacy or ethical restrictions.
