# Supplementary material for: Super‐Enhancer Reprograming Driven by SOX9 and TCF7L2 Represents Transcription‐Targeted Therapeutic Vulnerability for Treating Gallbladder Cancer
Source: Adv Sci (Weinh). 2024 Nov 4;11(47):2406448. doi: 10.1002/advs.202406448 (PMC11653766; doi:10.1002/advs.202406448)
Supplement: Supplementary file 1 — Supporting Information [file ADVS-11-2406448-s004.pdf]

ADVANCED SCIENCE
Open Access

Supporting Information

for *Adv. Sci.*, DOI 10.1002/advs.202406448

Super-Enhancer Reprograming Driven by SOX9 and TCF7L2 Represents Transcription-Targeted Therapeutic Vulnerability for Treating Gallbladder Cancer

Siyuan Yan, Zhaonan Liu, Teng Wang, Yi Sui, Xiangsong Wu, Jiayi Shen, Peng Pu, Yang Yang, Sizhong Wu, Shimei Qiu, Ziyi Wang, Xiaoqing Jiang, Feiling Feng, Guoqiang Li, FaTao Liu, Chaoxian Zhao, Ke Liu, Jiayi Feng, Maolan Li, Kwan Man, Chaochen Wang, Yujie Tang* and Yingbin Liu**

The PDF file includes:

Materials and Methods
Figure S1 to S13
Reference

Other Supplementary Material for this manuscript includes the following:

Table S1 to S4

Materials and methods

Ethics:

The Ethics Committee of Xinhua Hospital Affiliated to Shanghai Jiao Tong University School of Medicine approved all human tissue sampling (Approval No. XHEC-D-2015-169). All patients provided informed consent and the study was performed in accordance with the Declaration of Helsinki.

Patient-Derived Xenograft Model:

Tumor specimens were obtained from gallbladder adenocarcinoma patients with their informed consent. Fresh surgical tumor tissues (P0) were sectioned into ~5 mm^3 pieces and implanted subcutaneously into the flanks of male BALB/c nude mice under anesthesia with 10% chloral hydrate. Xenografts appeared at the graft site 2 to 3 months after grafting, and they were subsequently transplanted into subsequent generations. When the tumors reached about $100mm^3$, the mice were assigned randomly to different groups (n = 5).

Small-molecule compounds:

THZ1 (A8882) was purchased from APExBIO (USA), SY-1365(HY-128587), CT7001(HY-103712) was purchased from MedChemExpress (Shanghai, China). All molecules were dissolved in DMSO to a final concentration of 10 mM, and were diluted to a final DMSO concentration of <0.1% by volume in DMEM for cell culture experiments. Small molecules used for high-throughput screening were obtained from TargetMol company (Catalog No. L1200).

Plasmid construction:

For RNA interference (RNAi), two validated hairpins(sigma) were cloned into pLKO.1 (Addgene, #10878) by using AgeI and EcoRI site (Thermo Fisher Scientific), and a nontargeting shRNA was used as a negative control. For CRISPR interference (CRISPRi), sgRNA were designed using the online sgRNA design tool (https://www.benchling.com/), cloned into the lentiGuide-Puro (Addgene#52963) vector by using the BsmBI site (Thermo Fisher Scientific)[1]. Lenti-dCas9-KRAB-blast (Addgene, 89567) was used to express the dCas9-KRAB fusion protein. For constitutive expression, pCDH-CMV-3xHA-MCS-3xFlag-EF1-Puro and pCDH-CMV-3xHis-MCS-3xMyc-EF1-Puro was used to exogenously express the cDNA constructs. For inducible silencing, shRNA was cloned into Dox-inducible shRNA expression plasmids pLKO Tet-On (Addgene, 21915), which was engineered to be inducible by tetracycline or analogues (Tet-On) and produces tightly regulated induction of shRNA expression in the presence of doxycycline (0.2ug/ml). All target oligonucleotides used in this study were purchased from Sangon Biotech (Shanghai, China) and are listed in Table S4.

Lentivirus production and transduction:

The vector together with the other two viral packaging vectors (psPAX2 and

pMD2.G) were transfected into 293T cells with PEI MAX (2476S, Polysciences). The lentiviral containing medium was harvested, replaced 24h, 48h and 72h after transfection, then centrifuged to remove cell debris, and the supernatant was filtered. Lentiviral particles were concentrated with PEG6000(Sigma Aldrich) and resuspended in PBS.

For lentiviral infection, cells were transduced at an MOI of 5–10 for 48h, and stable cell lines were selected with the treatment of 2 μg/mL puromycin (YEASEN, 60210ES25) for another two days.

Chromatin immunoprecipitation and ChIP-seq library preparation:

ChIP-seq was performed as previously described. Briefly, 1×10^7 cells were formaldehyde fixed (1% final concentration) for 8 minutes at room temperature. The reaction was quenched by 0.125M glycine for 5 minutes and followed by cold PBS washes twice. The cells were digested by Micrococcal Nuclease (New England Biolabs, M0247S) for 10min at 37°C, and followed by sonication with the Active Motif EpiShear Probe Sonicator for 5 cycles (20% amplification, 20s on/30s off). Then 5–10g sheared chromatin samples were immunoprecipitated with 5μg H3K27ac antibodies (Active Motif, AM39133) overnight at 4°C while rotating. On the following day, the antibody-chromatin complex was added onto the Pierce ChIP-grade Protein A/G Magnetic Beads (Thermo Fisher Scientific, 26162) and incubated 4h at 4°C while rotating. Then the beads were washed three times with low salt buffer (50 mM Tris pH 7.4, 150 mM NaCl, 1% NP-40, 0.5% Sodium deoxycholate, 0.1% SDS) and once with high salt buffer (50 mM Tris pH 7.4, 500 mM NaCl, 1% NP-40, 0.5% Sodium deoxycholate, 0.1% SDS). The DNA bound to the antibody-conjugated beads was eluted with elution buffer and de-crosslinked by shaking at 1,200 rpm for 30 minutes at 65°C. De-crosslinked DNA was purified for further detection.

The ChIP-Seq library was prepared using ChIP-Seq DNA sample preparation kit (NEBNext® Ultra™II DNA) according to the manufacturer's instructions. For ChIP-seq, extracted DNA was ligated to specific adaptors followed by deep sequencing in the Illumina Novaseq 6000 using 150bp paired-end.

Cleavage Under Targets and Tagmentation (CUT&Tag):

CUT&Tag assay was performed as described previously with modifications[2]. As for frozen tissue, native nuclei were purified from frozen samples as previously described[3]. 500000 nuclei were washed twice gently with wash buffer (20 mM HEPES pH7.5; 150 mM NaCl; 0.5 mM Spermidine; 1× Protease inhibitor cocktail). As for cells, 100000 cells were directly washed twice gently with wash buffer. 10μl concanavalin A coated magnetic beads (Bangs Laboratories) were added per sample and incubated at RT for 10min. Remove unbound supernatant and resuspended bead-bound cells with dig-wash-buffer (20 mM HEPES pH 7.5; 150 mM NaCl; 0.5 mM Spermidine; 1× Protease inhibitor cocktail; 0.05% Digitonin; 2 mM EDTA) and a 1:50 dilution of primary antibody or IgG control antibody (normal rabbit IgG: Millipore cat.no. 12-370, normal mouse IgG: Millipore cat.no. 12-371) incubated on a rotating platform overnight at 4°C. The primary antibody was removed using magnet stand. Secondary

antibody (Anti-Rabbit IgG antibody, Goat monoclonal: Millipore AP132) was diluted 1:100 in dig-wash-buffer and cells were incubated at RT for 60 minutes. Cells were washed using the magnet stand 2-3 times in dig-wash-buffer. A 1:100 dilution of pA-Tn5 adapter complex was prepared in dig-med buffer (0.01% Digitonin; 20 mM HEPES pH7.5; 300 mM NaCl; 0.5 mM Spermidine; 1× Protease inhibitor cocktail) and incubated with cells at RT for 1h. Cells were washed 2–3 times for 5 minutes in 1ml Dig-med buffer. Then cells were resuspended in tagmentation buffer (10 mM MgCl2 in Dig-med Buffer) and incubated at 37°C for 1h. DNA was purified using phenol-chloroform-isoamyl alcohol extraction and ethanol precipitation.

To amplify libraries, 21μl DNA was mixed with 2μl of a universal i5 and a uniquely barcoded i7 primer. A volume of 25 μl NEBNext HiFi 2× PCR Master mix was added and mixed. The sample was placed in a Thermocycler with a heated lid using the following cycling conditions: 72 °C for 5 min (gap filling); 98 °C for 30 s; 14 cycles of 98 °C for 10 s and 63 °C for 30 s; final extension at 72 °C for 1 min and hold at 8 °C. libraried clean-up was performed XP beads (Beckman Counter).

The size distribution of libraries was determined by Agilent 4200 TapeStation analysis, and libraries were mixed to achieve equal representation as desired aiming for a final concentration as recommended by the manufacturer. Sequencing was performed in the Illumina Novaseq 6000 using 150bp paired-end following the manufacturer’s instructions.

Co-Immunoprecipitation:

Protein samples were washed with PBS then harvested and lysed directly in NETN150 buffer (NaCl 150 mM, EDTA 1 mM, Tris 20 mM pH 7.6, NP40 1%). Lysates were incubated with HA-beads /myc-beads (B26302/B26202, bimake, USA) with rotation at 4°C after quantified. Beads were washed by NETN150 lysis buffer for 3 times. Proteins were released from beads after denatured and boiled, then separated with SDS-PAGE for immunoblotting.

Small-molecule sensitivity profiling and validation:

Drugs library screening was performed using drug screening platform of Shanghai Ruijin Hospital. NOZ, GBC-SD, JXQ-3D-4494 and JXQ-3D-902R2 cells were each seeded overnight in 384-well (Corning) microtiter plates at a density of 750, 500, 750 and 750 cells per well, respectively. The following day, compound or DMSO was added to wells. Cells were incubated at 37 °C, and cell viability was assayed 3 days after the addition of compound or DMSO using the CellTiter-Glo reagent (Promega). For each cell line, primary screening was performed once with two replicates.

For validation, NOZ, GBC-SD, JXQ-3D-4494, JXQ-3D-902R2, JXQ-3D-4786, L-2F7, RPE-1 cells were seeded overnight at a concentration of 1000 cells per well in 96-well plates. The following day, compound or DMSO was added to wells using an Picus Electronic Pipette. Each compound was tested using eight concentrations, in quadruplicate (four wells treated in parallel). Cell viability was assayed 3 days after compound addition with the CellTiter-Glo reagent (Promega).

The sensitivity of each cell line to a tested compound was quantified as follows:

at each compound concentration, the luminescence value of quadruplicate was normalized to vehicle (DMSO) treatment to generate an inhibition rate. Curves were fit with nonlinear sigmoid functions, and the IC50 for each cell line-compound pair was calculated by numerically integrating under the eight-point concentration-response curve.

Immunohistochemistry:

Tumor xenografts were collected and fixed overnight in 4% paraformaldehyde, washed, embedded in paraffin, and sectioned. IHC and HE staining were performed as described previously. Briefly, endogenous peroxidases were inactivated by 3% hydrogen peroxide. Nonspecific signals were blocked for 30 minutes using 3% bovine serum albumin. Tumor samples were stained with the following primary antibodies: Ki67(Abcam, ab16667, 1:200), Cleaved-Caspase3(CST, 9661s, 1:500), TCF7L2(CST, 2569s, 1:100), SOX9(Millipore, A5535, 1:1000). After overnight incubation, the slides were washed and incubated with secondary antibody (HRP-Polymer) for 50 minutes at room temperature. The slides were washed three times and stained with 3,3'-diaminobenzidine (DAB) substrate (G1211, Servicebio). The slides were then counterstained with hematoxylin and mounted with mounting medium. Histological sections were reviewed using the Aperio ImageScope Viewer. For each slide, five fields were picked randomly and the staining intensity was quantified by ImageJ software (v1.53)[4].

To measure the immunoreactivity of different markers in patient cohort, the pathologists who as blinded to patients' outcomes performed the image analysis based on the staining of density and intensity. The scores were as follows: 0 for 0%–25% density or negative intensity, 1 for 26%–50% density or weak intensity, 2 for 51%–75% density or medium intensity, and 3 for 76%–100% intensity or strong intensity. The final semi-quantitative score = (density score+1) × (intensity score+1), ranging from 1 to 16.

Animal study:

All animal study was approved by Institutional Animal Care and Use Committee of Shanghai Jiao Tong University School of Medicine (registry number. JUMC-2023-184-A) and conducted according to ARRIVE guidelines. For subcutaneous tumor growth assays, 1 x 10^6 cells in 100µL of 1:1 DMEM/Matrigel Matrix (354230, Corning) solution were injected into each flank of 5-7-week-old athymic nude male mice (Shanghai Experimental Animal Center of the Chinese Academy of Sciences, Beijing, P.R. China). For PDX model, the tumor fragment (3 mm^3) was planted into flanks of nude mice. Tumor growth was followed by caliper measurement. Tumor volume (V) was calculated using the equation $V = (\text{length} \times \text{width}^2) \times 0.5$. When the tumor volume approached to 100 mm^3, mice were randomly divided into two groups. For THZ1 treatment in vivo, two groups were then treated with vehicle (DMSO in 5% dextrose), THZ1 (20mg/kg in vehicle solutions) intraperitoneally twice daily. For in vivo TET-ON inducible SOX9 or TCF7L2 knockdown in intraperitoneally xenograft mouse model, experimental group mice were fed with doxycycline (0.35% w/v)

containing 5% sucrose water. Control mice were fed with 5% sucrose water only. Tumor volume was measured every 2–3 days. The maximum permitted endpoint of a mean diameter of 12 mm for a superficial tumor was not exceeded in any of the experiments. Upon harvesting tumors, tumors were cut 3 parts, with one-third fixed in formalin for histopathology analysis, one third fixed in RNA Later for qRT-PCR analysis, and the rest fast-frozen for WB.

mRNA and protein detection:

Total RNA was extracted with TRIzol Reagent (15596018, Invitrogen) as per the manufacturer's protocol and 450ng RNA was used to generate cDNA using the RevertAid Master Mix (M1631, Thermo Fisher Scientific), quantitative PCR performed using PowerUp SYBR Green Master Mix (A25742, applied biosystems) on the Applied Biosystems QuantStudio Real-Time PCR System. Total cDNA of Drosophila S2 cells, serving as spike-in reagent, was added to total cDNA with mass ratio of 1:10. All assays were performed in triplicate, and relative gene expression were normalized to housekeeping genes. Primer sequences are listed in Table S4.

Total protein was extracted from cell pellets using RIPA buffer (Tris (pH7.4) 50 mM, NaCl 150 mM, NP-40 1%, sodium deoxycholate 0.50%, sodium dodecyl sulfate 0.10%, Protease inhibitor Cocktail 1X, Phosphatease inhibitor Cocktail 1%). Protein concentration was quantified with Pierce BCA Protein Assay (23225, Thermo Fisher Scientific). Proteins were separated by SDS-PAGE, transferred onto PVDF membrane (Millipore) and blotted with TCF7L2(CST, 2569s, 1:100), SOX9(Millipore, 702016, 1:100), GAPDH (60004-1-Ig, Proteintech, 1:5000) antibodies. Secondary antibodies were horseradish peroxidase–conjugate goat anti-rabbit/mouse IgG (0.2 mg/mL; Pierce, 31460 or 31430). Chemiluminescence were detected by Luminescent Image Analyzer (Fujifilm, LAS-4000) after incubation with enhanced ECL (E412, Vazyme).

Luciferase reporter assay:

Enhancer activity was measured by luciferase reporter assays. The regions comprising the SOX9 super-enhancer peak (approximately 350 bp) was cloned into the pGL4.27 Luciferase Reporter Vectors (Promega plasmid #E8451) containing a Firefly luciferase. Genomic DNA from NOZ cells was used as template, primers used are listed in Table S4. NOZ, L-2F7 and 293T cells were seeded into 24-well plates, and co-transfected with 500 ng of enhancer reporter plasmid and 10 ng of SV40 Renilla luciferase vector (Promega E6911) as a normalization control using ViaFect™ Transfection Reagent (Promega). After 48 hours of transfection, luciferase activity was measured using the Dual Luciferase Reporter Assay Kit (Vazyme, DL101-01).

RNA-seq:

Total RNA was extracted from cells in three replicates using the Trizol reagent according to the manufacturer's protocols. RNA degradation and contamination was monitored on 1% agarose gels. RNA purity was checked using the NanoPhotometer® spectrophotometer (IMPLEN, CA, USA). RNA concentration was measured using Qubit® RNA Assay Kit in Qubit®2.0 Flurometer (Life Technologies, CA, USA). RNA

integrity was assessed using the RNA Nano6000 Assay Kit of the Bioanalyzer-2100 system (Agilent Technologies, CA, USA).

A total amount of 3μg RNA per sample was used as input material for the RNA sample preparations. Sequencing libraries were generated using NEBNext® UltraTM RNA Library Prep Kit for Illumina® (NEB, USA) following manufacturer's recommendations and index codes were added to attribute sequences to each sample. Briefly, mRNA was purified from total RNA using poly-T oligo-attached magnetic beads. Fragmentation was carried out using divalent cations under elevated temperature in NEBNext First Strand Synthesis Reaction Buffer (5). First strand cDNA was synthesized using random hexamer primer and M-MuLV Reverse Transcriptase (RNase H-). Second strand cDNA synthesis was subsequently performed using DNA Polymerase I and RNase H. Remaining overhangs were converted into blunt ends via exonuclease/polymerase activities. After adenylation of 3' ends of DNA fragments, NEBNext Adaptor with hairpin loop structure were ligated to prepare for hybridization. In order to select cDNA fragments of preferentially 250~300bp in length, the library fragments were purified with AMPure XP system (Beckman Coulter, Beverly, USA). Then 3 μl USER Enzyme (NEB, USA) was used with size-selected, adaptor-ligated cDNA at 37°C for 15 minutes followed by 5 minutes at 95°C before PCR. Then PCR was performed with Phusion High-Fidelity DNA polymerase, Universal PCR primers and Index (X) Primer. At last, PCR products were purified (AMPure XP system) and library quality was assessed on the Agilent Bioanalyzer 2100 system.

Pooled CRISPR-Cas9 screening:

Our sgRNA library contained two sublibraries, with sublibrary #1 containing 5648 sgRNAs [5517 sgRNAs targeting 910 epigenetic/transcriptional factor genes and 131 nontargeting control (NTC) sgRNAs] and sublibrary #2 containing 1012 sgRNAs (992 sgRNAs targeting 199 epigenetic/transcriptional factor genes and 20 NTC sgRNAs).

CRISPR-Cas9 screening was performed as previously described.[5] Briefly, the GBC cell lines, NOZ and GBC-SD, were first transduced with lentiCas9-Blast (52962, Addgene) at a low multiplicity of infection (MOI<0.7) to generate cell lines with stable Cas9 expression. The lentiviral sgRNA library targeting epigenetic-related genes was produced using HEK293T cells as described above, and was transduced into approximately 10 million Cas9-expressing cells at a low MOI (<0.3) to ensure that >85% of cells had a single sgRNA integration, resulting in at least 500× sgRNA representation. Two days after sgRNA library transduction, cells were subjected to puromycin selection (NOZ 1 mg/mL, GBC-SD 4 mg/mL) for 5 d. Considering that only 10% of the initial population survives allograft transplantation in vivo and to ensure 500× coverage of the sgRNA library for in vivo screening, 3×10^7 cells were subcutaneously injected into the dorsal flanks of 10 mice. Further, 10 million transduced cells were cultured in vitro. Both in vitro and in vivo screenings were simultaneously terminated after 14 population doubling times. At least 10 million cells were harvested at the beginning and end of the assay. Genomic DNA was extracted using the FastPure Cell/Tissue DNA Isolation Mini Kit (DC102-01, Vazyme).

sgRNA sequences were amplified using the 2× KAPA HiFi HotStart ReadyMix (KK2602, Roche). PCR products were subjected to next-generation sequencing by Novogene Technology.

Colony formation assays:

To determine colony viability, 1000 cells were seeded on 6-well plates and incubated for 14 days when colonies with more than 50 cells formed. The colonies were fixed with 4% formaldehyde for 15 minutes and then 0.05% crystal violet for 15 minutes. After that, plates were washed with PBS for 3 times and then captured.

Immunofluorescence:

Cells were plated on 13mm coverslips (VWR, 631-0149) in 24-well plates and cultured overnight. The cells were then washed with PBS, fixed with 4% paraformaldehyde for 15 minutes, treated with 0.1% Triton X-100 for 15 minutes, and blocked with 5% BSA for 1h at room temperature. The cells were incubated with primary antibodies diluted in 1% BSA (Abclonal, A19657, 1:200) in a wet box at 4℃ overnight. After washing in PBS, cells were then incubated with fluorescent conjugated secondary antibody (Thermo Fisher, A11008) diluted in 1% BSA (1:500) in a wet box at room temperature for 1h. After washing in PBS, cells were finally incubated with 1 ug/ml DAPI (Sigma, D9542) for 5 min. The coverslips were covered with ProLong™ Diamond Antifade Mountant (Thermo Fisher, P36965) on the slides and observed by Confocal Microscopes (OLYMPUS, FV3000).

Sphere formation assay:

Cells were suspended in the corresponding complete culture medium, and cultured in 96-well Clear Flat Bottom Ultra-Low Attachment Microplate (Corning, 3474) at 5% CO_2, 37℃ for 7 days. Sphere numbers in each well were counted, and the cell viability was examined by CellTiter-Glo assays.

Multiplex immunohistochemistry:

Tumor tissue samples were procured and processed through formalin fixation and paraffin embedding (FFPE). 5mm sections were prepared and subjected to antigen retrieval using a pH 9.0 EDTA buffer in a microwave oven. Non-specific binding was minimized by blocking with a 10% normal serum. Primary antibodies were applied overnight at 4°C, followed by Opal polymer horseradish peroxidase (HRP)-conjugated secondary antibodies (Akoya Biosciences, ARH1001EA). Tyramide signal amplification was performed using the Opal 7-color TSA kit, according to the manufacturer's protocol, with sequential antigen retrieval and washes to prevent bleed-through between channels. The primary antibodies and corresponding TSA dyes are listed: SOX9 (Merck, AB5535, 1:2000) with Opal570, TCF7L2 (CST, 2569, 1:200) with Opal520, CK19 (Santacruz, sc-6278, 1:900) with Opal480, P21 (CST, 2947S, 1:100) with Opal620.The sections were then counterstained with DAPI and scanned using a multispectral imaging system (Akoya Biosciences, PhenoImager HT) to visualize and quantify the immunofluorescence.

For quantitative image analysis, the QuPath-0.5.1 software was utilized to detect, measure, and classify cells based on their immunofluorescent image. Tissue sections were manually annotated by a certified oncopathologist to identify tumor regions, with non-neoplastic tissues excluded from further analysis. Within these tumor areas, cells were automatically detected utilizing the DAPI nuclear signal with default algorithmic parameters. Cellular features were extracted from each fluorescence channel, and this information was subsequently input into a machine-learning algorithm to classify cells as positive or negative for the specific markers. CK19, a recognized marker for cancer cells, was employed to identify malignant cells. These cancer cells were then further categorized into distinct groups based on their SOX9 and TCF7L2 expression patterns.

Data processing：

CRISPR screening analysis:

The MAGeCK and MAGeCKFlute software packages were used to analyze the genomic DNA-derived CRISPR–Cas9 screens[6, 7]. MAGeCK mapped the sequencing reads to a reference library of sgDNA sequences and returned the number of reads that match the sgDNA sequences without allowing mismatches. Each sample was then evaluated based on the Gini index, missed gDNAs, and correlation between replicate sgDNA counts. Significantly enriched or depleted genes under test or control conditions were detected using the robust rank aggregation method in MAGeCK.

RNA-seq analysis:

Trim Galore! was used to automatically detect and trim adapters[8]. Sequencing reads were mapped to hg38 using Hisat2[9]. Read counts were generated using HTSeq (v0.11.1)[10]. Differentially expressed genes were identified using DESeq2 (v1.34.0)[11]. Gene Ontology and Gene Set Enrichment analyses(GSEA) was performed using the R package, ClusterProfiler (4.2.2)[12].

H3K27ac ChIP-seq / CUT&Tag data analysis:

H3K27ac and input ChIP-seq reads were aligned to the hg38 human genome using bowtie2(v2.5.0)[13]. BAM files were processed using SAMtools (v1.15.1)[14]. H3K27ac peaks were called using MACS2 (v2.2.7.1) with a ChIP input file at q-value cutoff of 0.01[15] as a control. BIGWIG track coverage files were generated from merged BAM files using the DeepTools (v3.3.1)[16] bamCoverage command with RPKM normalization. Genomic coverage heatmaps were generated using the DeepTools “computeMatrix” and “plotHeatmap” functions or the Integrative Genomics Viewer(IGV)[17]. SEs were called using ROSE2 on the hg38 human genome with a stitching distance (-s) of 12500 bp and a transcription start site exclusion distance (-t) of 2500 bp[18, 19]. SEs were ranked by counting the H3K27ac signal in the ChIP file compared with that in the matched input file, and annotated to the closest gene (FPKM > 1).

A typical enhancer and SE correlation analysis was performed using DiffBind (v3.8.1)[20]. Accounting for sample heterogeneity, we defined GBC SEs as those identified in at least 50% of tumor samples, and the CC SEs were those presented in both CC samples' predictions. GBC-specific SEs were characterized as those within GBC SEs that exhibited less than 50% overlap with CC SEs. Super-enhancers were linked to the closest gene by the ROSE algorithm. Motifs were called from GBC-specific SE regions in the hg38 genome using the HOMER "findmotifsgenome.pl" script[21]. Enriched ontology analysis of colocalized binding sites was performed using the Genomic Regions Enrichment of Annotations Tool (GREAT)[22, 23]. The CRC TFs were then identified using the "coltron" algorithm (https://pypi.org/project/coltron)[24, 25].

TF CUT&Tag data analysis:

Qualified reads were aligned to the hg38 human genome using Bowtie2 (2.2.5) with the following parameters: --phred33 -× GRCh38.p13. genome –threads=4 --no-unal --end-to-end --sensitive. MACS2 was used for peak calling using the following parameters: macs2 callpeak -t IgG.bam -f BAM -g hs -n name --scale-to large -B -q (X 0.005, Y 1×10-8). The "annotatePeaks.pl" script from the Homer software suite was used for annotation. Depositions along genomic regions were visualized using IGV (2.6.3). Read counts were normalized by RPKM computed in each 10bp bin among defined regions, and then used to generate heatmaps using DeepTools (v3.3.1). BEDtools (v2.30.0) was used to identify differential binding sites[26]. Hypergeometric Optimization of Motif Enrichment (HOMER) analysis of differential binding sites was performed to identify enriched TF motifs.

Hi-C data analysis:

As previously described, Hi-C data were generated, processed, and analyzed using the HiC-pro pipeline (v3.0.0) [27]. Reads were binned at a 5-kb resolution and converted to a .hic file using the "hicpro2juicebox.sh" script, visualized using the "plotgardener" package (v1.4.1) [28]. The contact matrix was then transferred to a .5k cool file using "hicConvertFormat" in HiCExplorer (v3.0) [29-31]. Loop domains were identified at a 5-kb resolution using Peakachu (v2.0) [32]. Based on the contact matrix depth, Peakachu suggested the most appropriate pretrained model and accordingly predicted the interaction loop. Significant loops were determined at probability score of 0.98 to reduce the number of false-positive loop calls. Default settings were used for all other parameters.

Single-cell RNAseq data analysis:

We reanalyzed our previously published 10x Genomics single-cell RNA-seq data from primary GBC adenocarcinoma tumor samples [33]. The data were aligned with Cell Ranger against the GRCh38. For quality control, samples with fewer than 1000 cells were excluded. Low-quality cells (detected genes per cell <= 500 or proportion of mitochondrial gene count per cell >= 10%) and potential doublets (detected genes per cell >=5000~7000) were filtered out. After quality control, samples from different patients were integrated using the FindIntegrationAnchors and IntegrateData

functions (Seurat V3)[34]. The integrated data were further processed in Seurat for dimension reduction and unsupervised clustering. Clusters were annotated based on the specific expression pattern of canonical genes identified by the Wilcoxon rank-sum test. Based on the initial cell-type identification, all the epithelial cells were extracted and inferred for CNVs in each cell, with T cells as the reference and spike-in. After K-means clustering of the CNV profiles, malignant epithelial cells were identified as those with chromosomal alterations (deletions or amplifications), and not in the same cluster with spike-in cells. GSVA analyses of pathways integrated with MsigDB and self-defined gene sets were conducted using GSVA (1.44.5)[35]. We inferred the activity of transcription factors (TFs) for each cluster using DoRothEA (1.7.2)[36]. CellCycleScoring function of Seurat was used to infer the cell cycle phase. CytoTRACE[37], based on the observation that transcriptional diversity—the number of genes expressed in a cell—decreases during differentiation, was applied to infer the differential state of the cells. The CIBERSORTx[38] (https://cibersortx.stanford.edu/) deconvolution algorithm estimated the abundance of 2 malignant epithelial cell subclusters among dbGAP (phs001404.v1. p1) late-stage GBC patients. Survival analysis between patient groups divided by single-cell malignant epithelial cell signature score was conducted using Kaplan-Meier methods and the log-rank test.

TCGA RNA-seq integrative Analysis:

Bulk RNA-seq data of gallbladder cancer tissues (EGAS00001003004) were integrated with TCGA using Toil[39] to reduce computational batch effects and create a consistent dataset.

Statistics:

The GBC patients were categorized into high and low groups based on the final IHC scores of the markers. Subsequently, Kaplan-Meier survival analysis was performed using the 'surviminer' and 'survival' R packages. Univariable Cox proportional-hazard models were used to obtain hazard ratios (HRs) with 95% confidence intervals (CIs). A multivariable Cox proportional hazards model was used to determine the independent prognostic factor associated with RFSs and overall survival.

Supplementary figures and legends

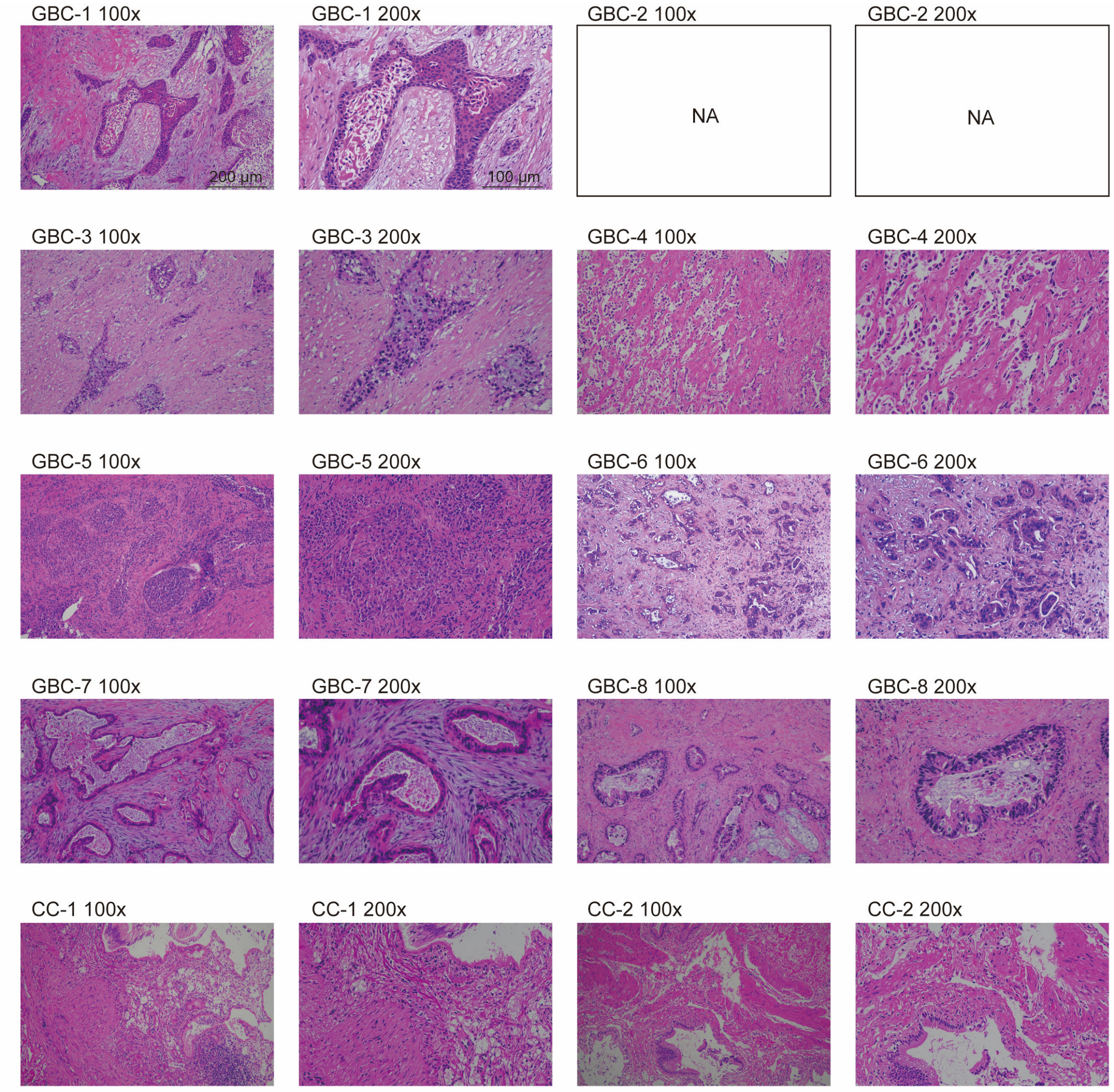

Figure S1 (related to figure 1).

Hematoxylin and eosin (H&E) staining of the samples for H3K27ac CUT&Tag (gallbladder cancer patients n = 8, chronic cholecystitis patients n = 2). Scale bars, 200 µm & 100 µm.

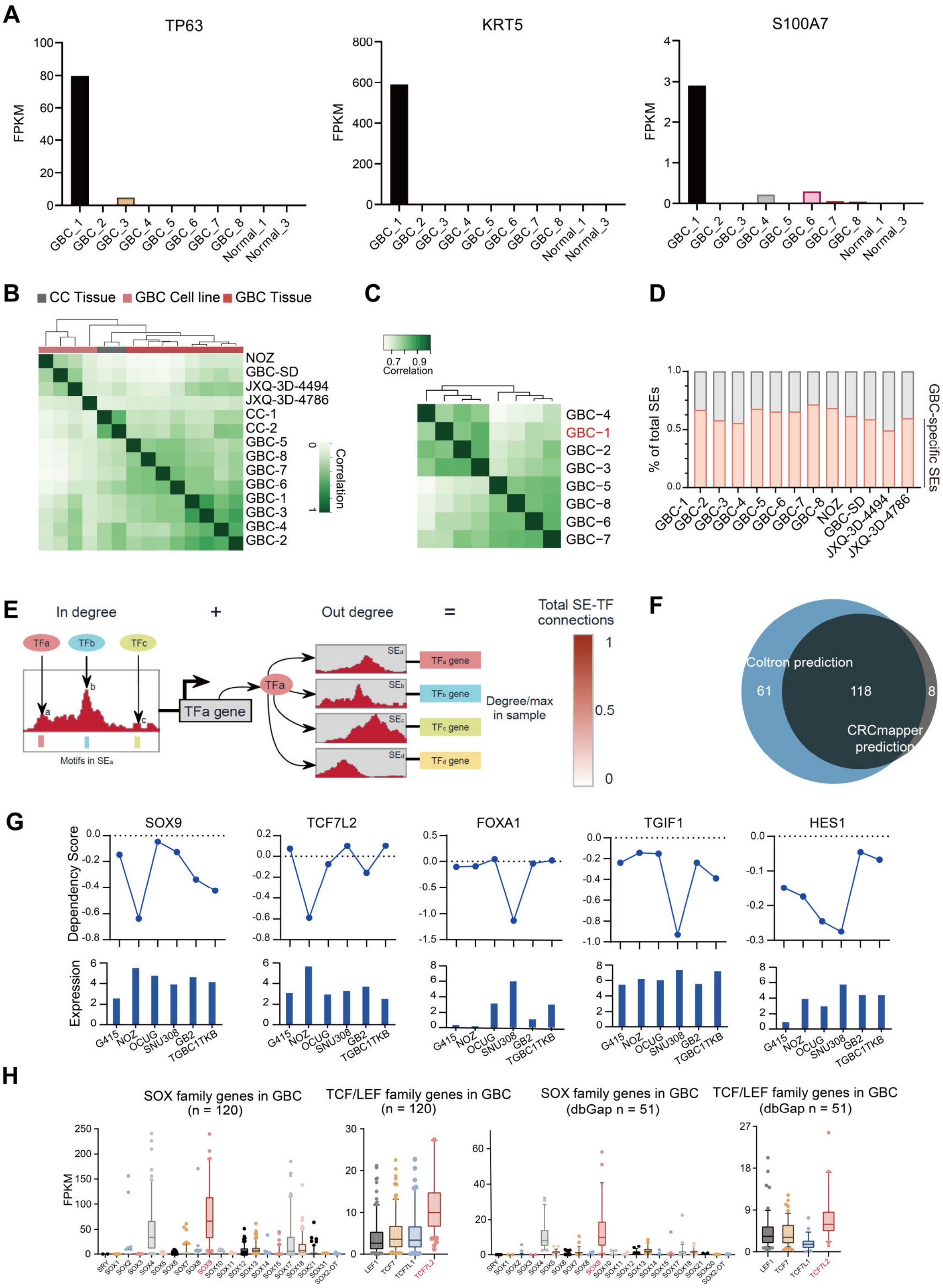

Figure S2 (related to figure 1).

A. Bar plot of SCC marker genes expression in each GBC sample.

B. Hierarchical clustering of the Pearson correlation coefficients among GBC tissue samples (n = 8), GBC cell lines (n = 4) and CC samples (n = 2) based on H3K27ac signal at typical enhancer regions.

C. Hierarchical clustering of the Pearson correlation coefficients among GBC tissue samples (n = 8) based on H3K27ac signal at super enhancer regions.

D. Bar plot of GBC-specific SE proportion (highlighted with red bars) in each GBC sample.
E. Schematic diagram for TF connectivity and CRC TFs identification. TFa and TFs elsewhere acting on the SE of TFa (in degree), modulate TFa gene expression and further acts on SEs of other genes (out degree). Total SE-TF connection equals sum of in degree and out degree connectivity, then scaled by dividing the maximal connection degree in the sample.
F. The number of CRC TFs predicted by COLTRON and CRCmapper calling algorithms.
G. The dependency score and expression of master TFs in 6 GBC cell lines collected in DepMap database.
H. Gene expression of all *SOX* and *TCF/LEF* family genes in GBC RNA-seq cohort (EGA database, n = 120 & dbGAP database, n = 51).

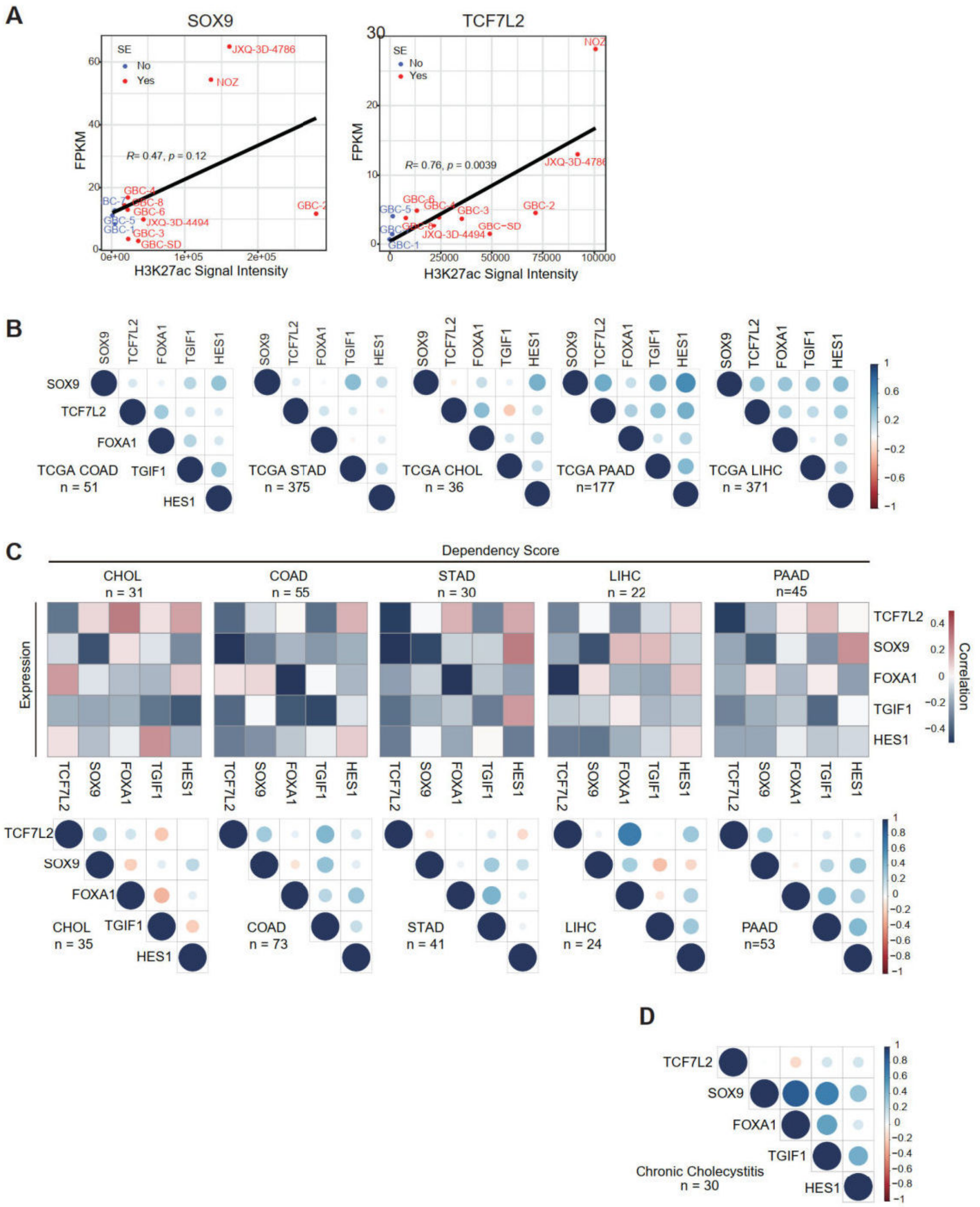

Figure S3 (related to figure 2).

A. Correlation analyses of mRNA level and H3K27ac signal intensity of SOX9 and TCF7L2.

B. Pearson correlation analysis of master TFs expression (FPKM) among primary cancer tissue samples in TCGA cholangiocarcinoma (CHOL, n = 36), colorectal cancer (COAD, n = 51), gastric cancer (STAD, n = 375), liver cancer (LIHC, n = 371), and pancreatic cancer (PAAD, n = 177). The area of circles show the absolute value of corresponding correlation coefficients.

C. Pearson correlation analysis of the dependency score and expression of master TFs among other cancer types cell lines (CHOL, n = 31; COAD, n = 73; STAD, n = 41; LIHC, n = 24; PAAD, n = 53) collected in DepMap database. The area of circles

show the absolute value of corresponding correlation coefficients.

D. Pearson correlation analysis of master TFs expression in benign CC tissue RNA-seq cohorts (n = 30). The area of circles show the absolute value of corresponding correlation coefficients.

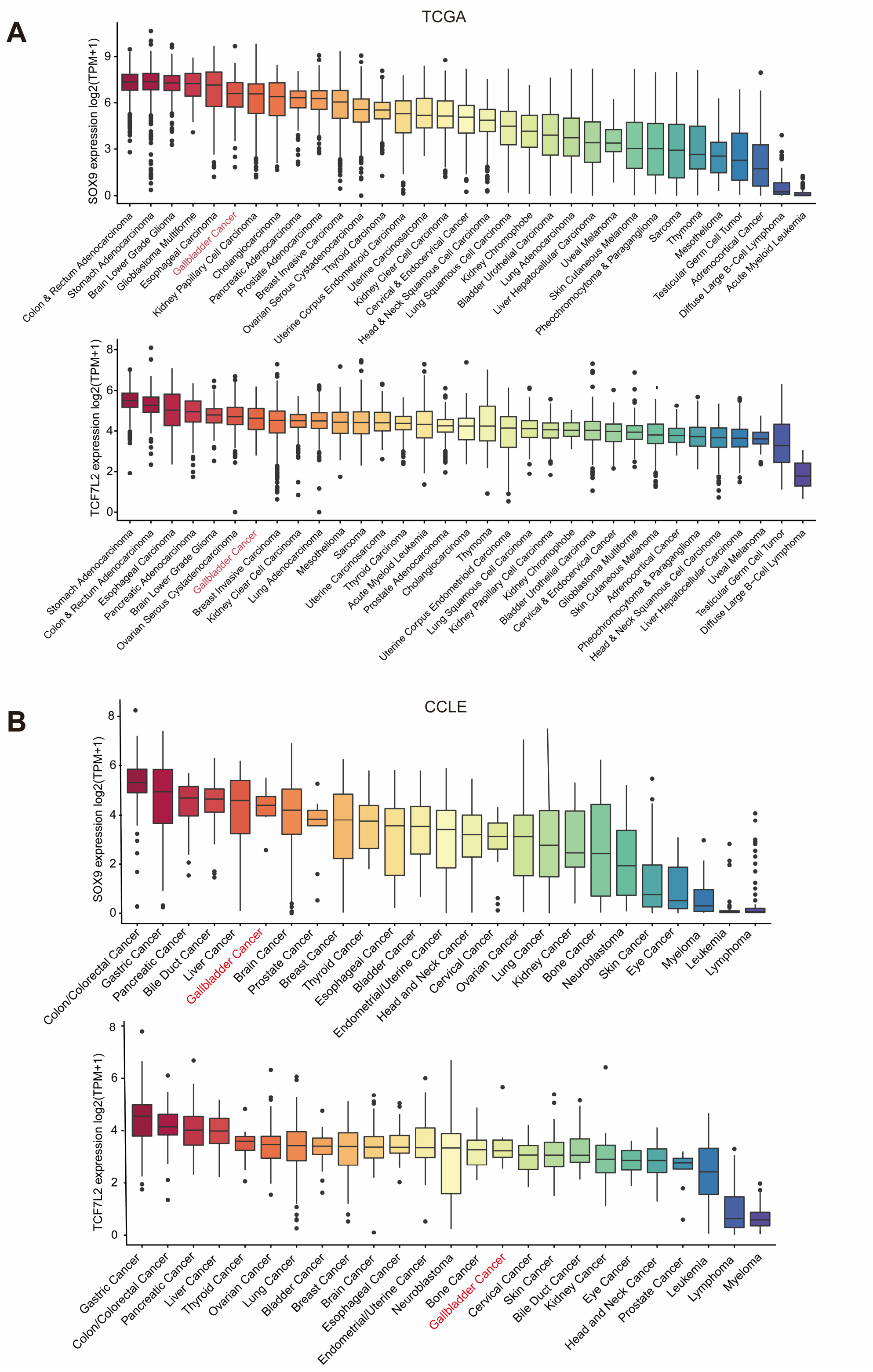

Figure S4 (related to figure 2).

A. Gene expression of SOX9 and TCF7L2 among gallbladder cancer (EGA database, n = 120) and other cancer types in TCGA database.

B. Gene expression of SOX9 and TCF7L2 among cell lines collected in CCLE database.

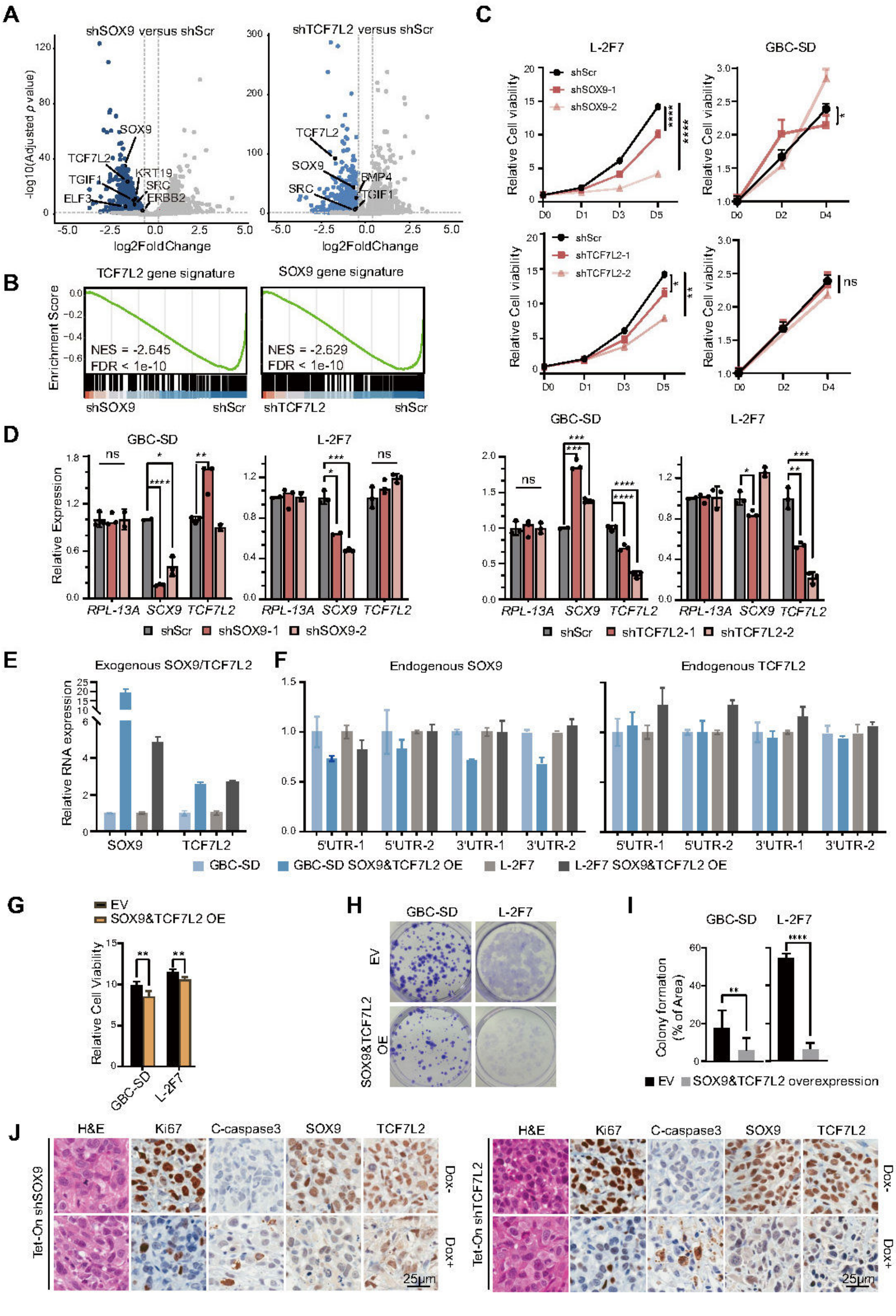

Figure S5 (related to figure 3).

A. Volcano plots showing differentially expressed genes after SOX9 (left) or TCF7L2 (right) silencing in NOZ cells. Genes with adjusted *p* value < 0.05 and log2FC < -0.4 are highlighted.

B. GSEA results of the TCF7L2 and SOX9 gene signature enrichment in NOZ cells with shSOX9 or shTCF7L2 versus shScr.

C. Cell viability in the GBC-SD and L-2F7 cells following knockdown with two independent, nonoverlapping shRNAs targeting SOX9 and TCF7L2 or a scramble shRNA. Two-way repeated measures ANOVA was used for statistical analysis with Dunnett multiple hypothesis test correction with five technical replicates.

D. The qPCR results of the coregulation between SOX9 and TCF7L2 in GBC-SD and L-2F7 cell line. Data are expressed as mean ± SD, unpaired t test was used for statistical analysis. ns, not significant, $*p<0.05$, $**p<0.01$, $***p<0.001$, $****p < 0.0001$.

E&F. Exogenous and endogenous mRNA expression levels of SOX9 and TCF7L2 in GBC-SD and L-2F7 cells with SOX9&TCF7L2 overexpression versus vehicle control.

G. CellTiter-Glo assays showing reduction of cell viability in 5 days after SOX9&TCF7L2 overexpression.

H&I. SOX9&TCF7L2 overexpression impaired the sphere-forming capacity of GBC-SD and L-2F7 cells.

J. Representative images of hematoxylin and eosin (H&E) and immunohistochemical staining for Ki67, cleaved-caspase3, SOX9 or TCF7L2 in tumor paraffin sections.

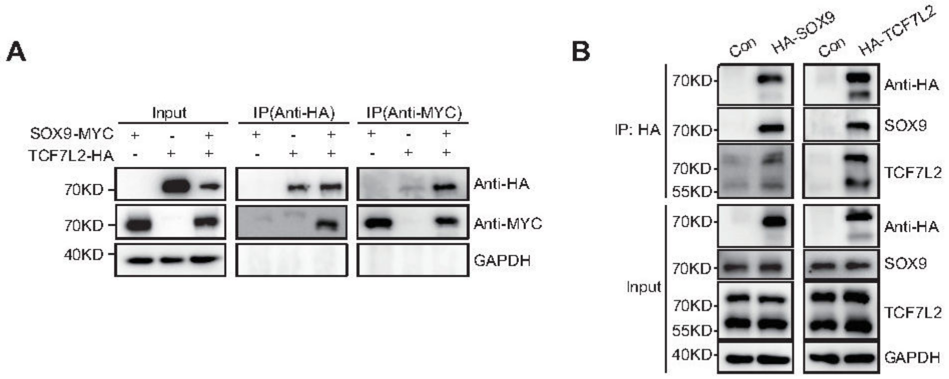

Figure S6 (related to figure 5).

A&B. Exogenous and endogenous SOX9-TCF7L2 protein interaction was demonstrated by co-immunoprecipitation assays in HEK293T cells (A) and NOZ cells (B).

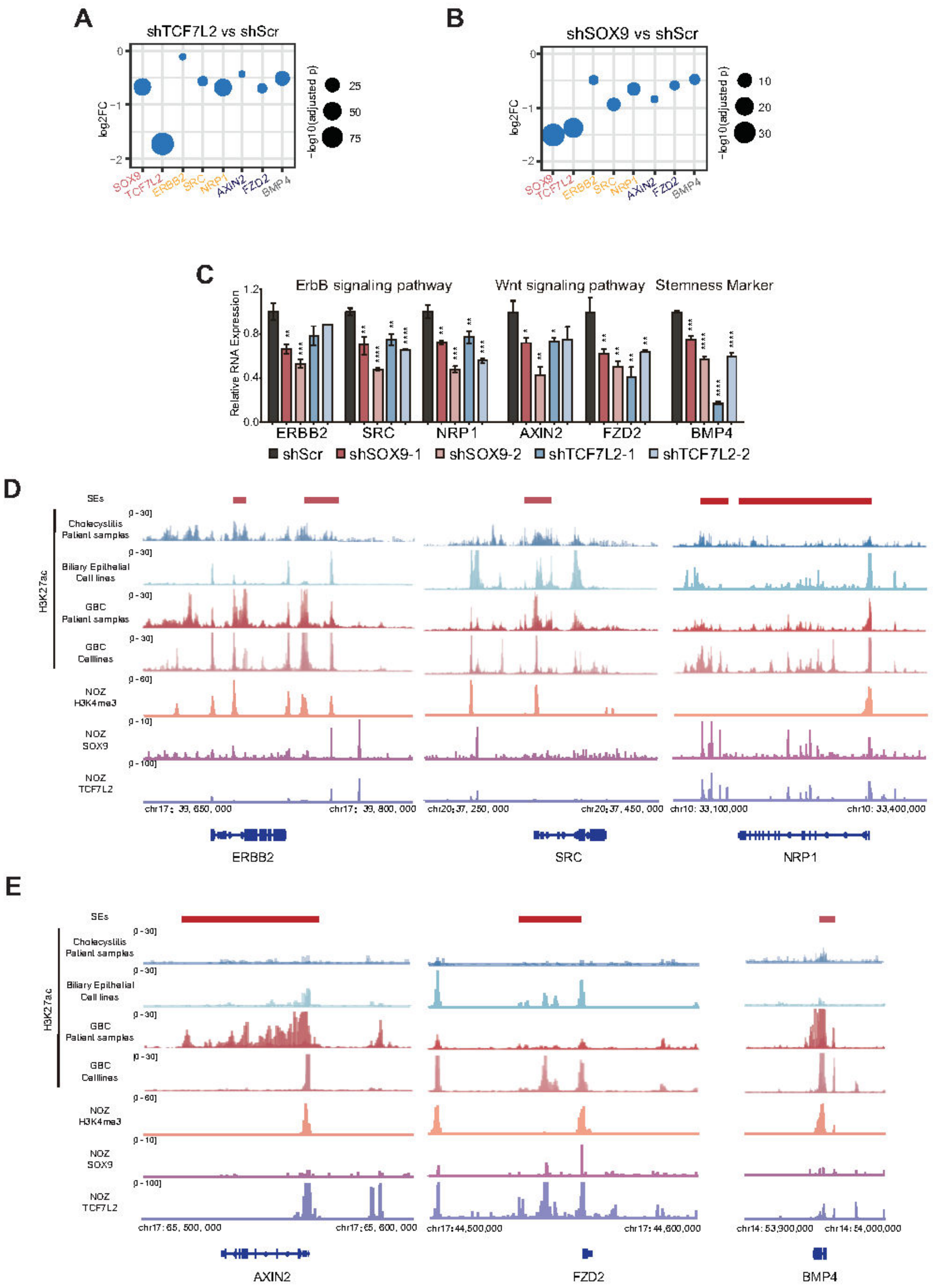

Figure S7 (related to figure 5).

A-C. mRNA levels of *SOX9*, *TCF7L2*, and the key target genes within the ErbB pathway (*ERBB2*, *SRC*, *NRP1*), Wnt signaling pathway (*AXIN2*, *FZD2*), and stem cell marker (*BMP4*) in NOZ cells with SOX9 or TCF7L2 knockdown by shRNA.

D-E. Genome tracks showing H3K27ac, H3K4me3, SOX9 and TCF7L2 CUT&Tag peaks at indicated genetic locus.

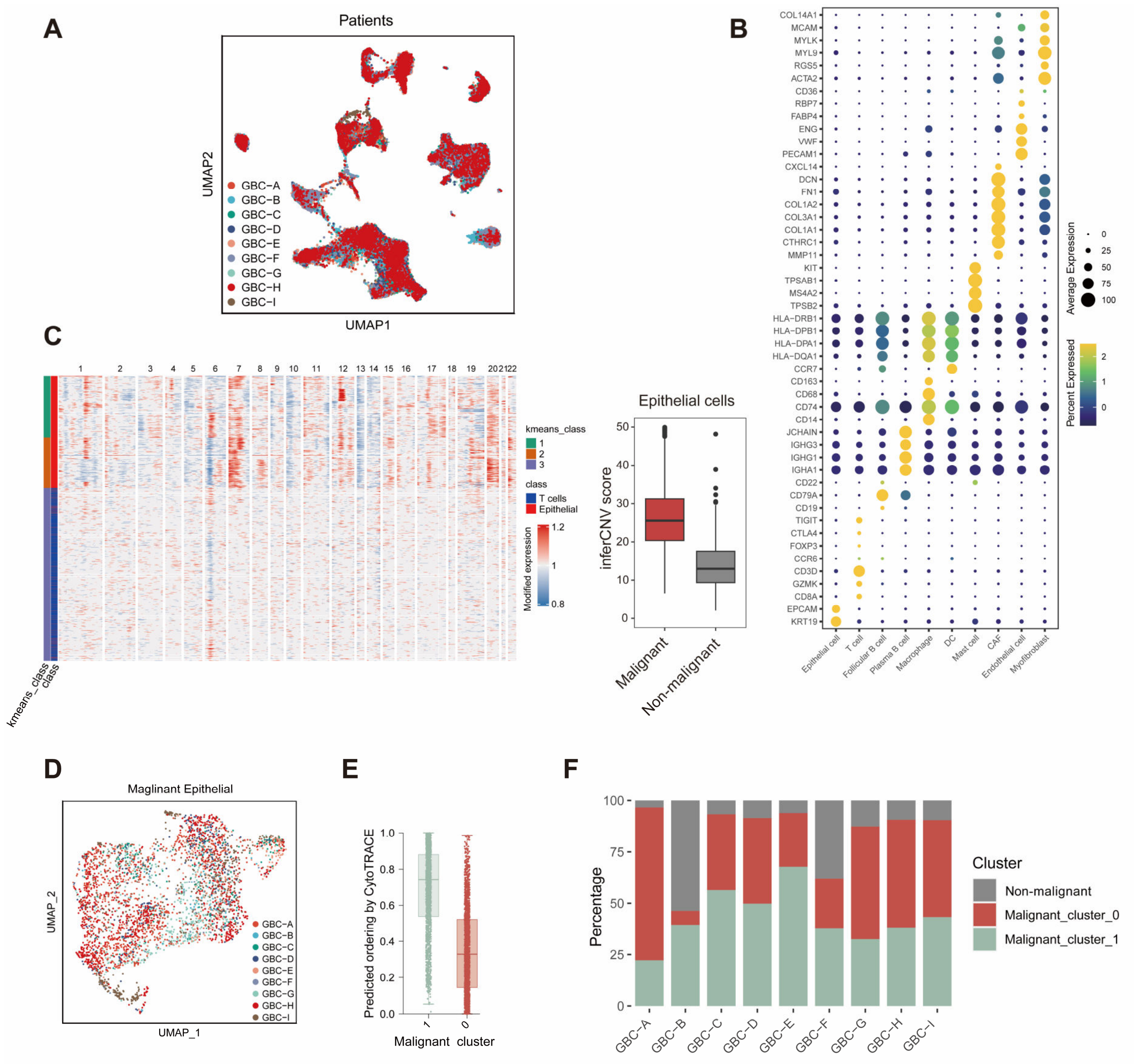

Figure S8 (related to figure 6).

A. UMAP visualization depicting the integrated scRNA-seq data of primary cancer tissues from 9 GBC patients, colored by different patients.

B. Bubble plots showing expression levels of marker genes for each cell type.

C. K-means clustering of the inferred copy number variations (CNVs) landscape analyzed with inferCNV for epithelial cells and reference T cells among all samples. The boxplot showing the inferCNV score of malignant and non-malignant epithelial cells.

D. UMAP plot visualizing malignant epithelial cell subclusters colored by sample.

E. Boxplot showing the degree of differentiation analyzed with CytoTRACE of two malignant epithelial cell subpopulations.

F. Bar plot showing the proportion of epithelial cell subpopulations within each sample.

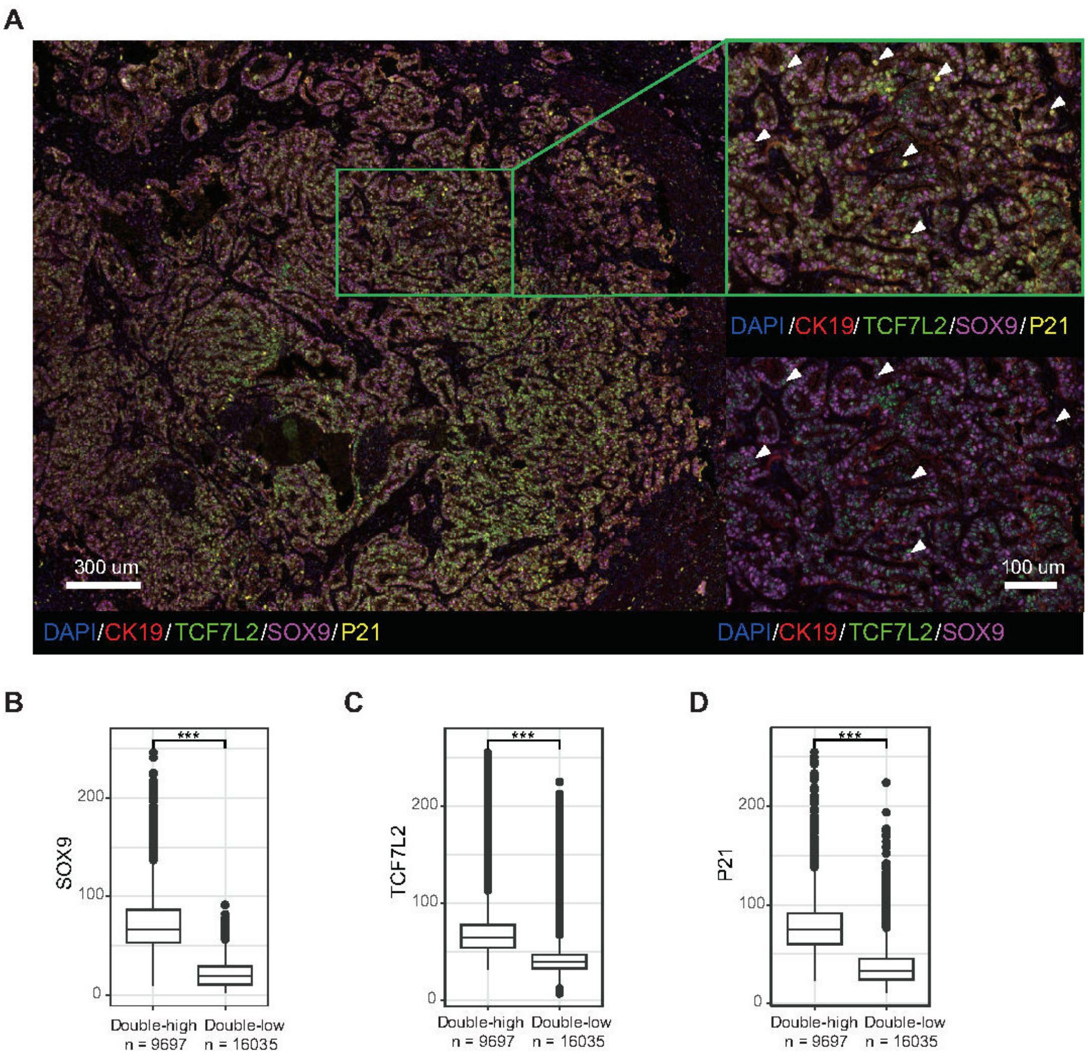

Figure S9 (related to figure 6).

A. Representative images (A) and quantification (B-D) from multiplex immunohistochemistry (mIHC) assessing the expression of SOX9, TCF7L2, CK19 and P21 in a representative GBC tissue sample. Unpaired *t* test was used for statistical analysis.

Variables	levels	stats	SOX9/TCF7L2 staining			
			Double-low (N=28)	Single-high (N=48)	Double-high (N=30)	*p* value
Age	Median (IQR)	68.00 (60.00 to 73.00)	64.50 (60.00 to 73.50)	69.00 (62.50 to 73.00)	64.00 (59.00 to 71.00)	.402
Sex	Female	58 (54.72%)	16 (57.1%)	26 (54.2%)	16 (53.3%)	.953
	Male	48 (45.28%)	12 (42.9%)	22 (45.8%)	14 (46.7%)	
Gallstone	No	53 (50.00%)	13 (46.4%)	23 (47.9%)	17 (56.7%)	.684
	Yes	53 (50.00%)	15 (53.6%)	25 (52.1%)	13 (43.3%)	
Tumor size (cm)	Median (IQR)	3.00 (2.00 to 5.00)	3.50 (2.25 to 7.50)	3.00 (2.00 to 4.50)	3.00 (2.00 to 4.00)	.244
T	T1	10 (9.43%)	2 (7.1%)	7 (14.6%)	1 (3.3%)	.274
	T2	39 (36.79%)	10 (35.7%)	16 (33.3%)	13 (43.3%)	
	T3	40 (37.74%)	13 (46.4%)	14 (29.2%)	13 (43.3%)	
	T4	17 (16.04%)	3 (10.7%)	11 (22.9%)	3 (10%)	
N	N0	49 (53.85%)	14 (56%)	24 (58.5%)	11 (44%)	.716
	N1	35 (38.46%)	10 (40%)	14 (34.1%)	11 (44%)	
	N2	7 (7.69%)	1 (4%)	3 (7.3%)	3 (12%)	
M	M0	81 (76.42%)	22 (78.6%)	38 (79.2%)	21 (70%)	.619
	M1	25 (23.58%)	6 (21.4%)	10 (20.8%)	9 (30%)	
Stage (AJCC 8th)	I-II	34 (35.42%)	9 (34.6%)	15 (34.9%)	10 (37%)	.978
	III-IV	62 (64.58%)	17 (65.4%)	28 (65.1%)	17 (63%)	
Histology	Others	23 (21.70%)	9 (32.1%)	10 (20.8%)	4 (13.3%)	.217
	Adenocarcinoma	83 (78.30%)	19 (67.9%)	38 (79.2%)	26 (86.7%)	
Differentiation	Grade I	5 (5.10%)	1 (4.3%)	4 (8.9%)	0 (0%)	.276
	Grade II	66 (67.35%)	16 (69.6%)	27 (60%)	23 (76.7%)	
	Grade III	26 (26.53%)	5 (21.7%)	14 (31.1%)	7 (23.3%)	
	Grade IV	1 (1.02%)	1 (4.3%)	0 (0%)	0 (0%)	
Liver metastasis	No	91 (85.85%)	24 (85.7%)	42 (87.5%)	25 (83.3%)	.876
	Yes	15 (14.15%)	4 (14.3%)	6 (12.5%)	5 (16.7%)	
Ki-67	Median (IQR)	40.00 (30.00 to 60.00)	40.00 (20.00 to 65.00)	40.00 (30.00 to 60.00)	40.00 (25.00 to 50.00)	.672
HER2	-	8 (32.00%)	1 (33.3%)	6 (40%)	1 (14.3%)	.330
	+	8 (32.00%)	2 (66.7%)	3 (20%)	3 (42.9%)	
	++	6 (24.00%)	0 (0%)	5 (33.3%)	1 (14.3%)	
	+++	3 (12.00%)	0 (0%)	1 (6.7%)	2 (28.6%)	
Surgical margin	R0	84 (79.25%)	24 (85.7%)	39 (81.2%)	21 (70%)	.303
	R1/R2	22 (20.75%)	4 (14.3%)	9 (18.8%)	9 (30%)	
Relapse in 1 year	No	27 (58.70%)	8 (80%)	16 (61.5%)	3 (30%)	.069
	Yes	19 (41.30%)	2 (20%)	10 (38.5%)	7 (70%)	

Figure S10 (related to figure 7).

Demographic and clinical characteristics of the GBC patients in the IHC cohort (n = 106).

Subgroup	stats	Hazard Ratio(95% CI)	*p* value
**Age**			
<=60	27 (25.5%)		
>60	79 (74.5%)	1.21 (0.70−2.11)	0.490
**Sex**			
Female	58 (54.7%)		
Male	48 (45.3%)	0.85 (0.53−1.37)	0.502
**Gallstone**			
No	53 (50.0%)		
Yes	53 (50.0%)	0.89 (0.55−1.44)	0.639
**Tumor size (cm)**			
<=3	53 (53.0%)		
>3	47 (47.0%)	1.59 (0.98−2.58)	0.062
**T**			
T1	10 (9.4%)		
T2	39 (36.8%)	0.80 (0.28−2.29)	0.681
T3	40 (37.7%)	3.51 (1.37−9.02)	0.009
T4	17 (16.0%)	4.51 (1.62−12.53)	0.004
**N**			
N0	49 (53.8%)		
N1	35 (38.5%)	2.97 (1.69−5.23)	<0.001
N2	7 (7.7%)	3.97 (1.69−9.34)	0.002
**M**			
M0	81 (76.4%)		
M1	25 (23.6%)	3.27 (1.96−5.46)	<0.001
**Stage (AJCC 8th)**			
I−II	34 (35.4%)		
III−IV	62 (64.6%)	4.70 (2.37−9.32)	<0.001
**Histology**			
Others	23 (21.7%)		
Adenocarcinoma	83 (78.3%)	0.92 (0.52−1.64)	0.788
**Differentiation**			
Grade I−II	71 (72.4%)		
Grade III−IV	27 (27.6%)	1.20 (0.69−2.08)	0.515
**Liver metastasis**			
No	91 (85.8%)		
Yes	15 (14.2%)	3.95 (2.15−7.27)	<0.001
**Surgical margin**			
R0	84 (79.2%)		
R1/R2	22 (20.8%)	4.28 (2.49−7.38)	<0.001
**SOX9&TCF7L2 staining**			
Double-low	28 (26.4%)		
Single-high	48 (45.3%)	1.04 (0.57−1.89)	0.907
Double-high	30 (28.3%)	2.12 (1.12−4.02)	0.022

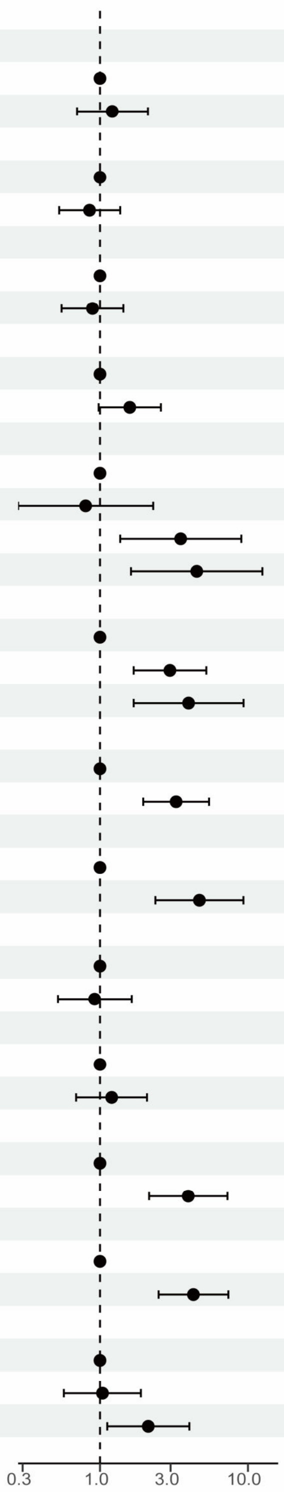

Figure S11 (related to figure 7).

Univariable Cox regression models to evaluate the association between clinicopathological factors and overall survival in the GBC cohort.

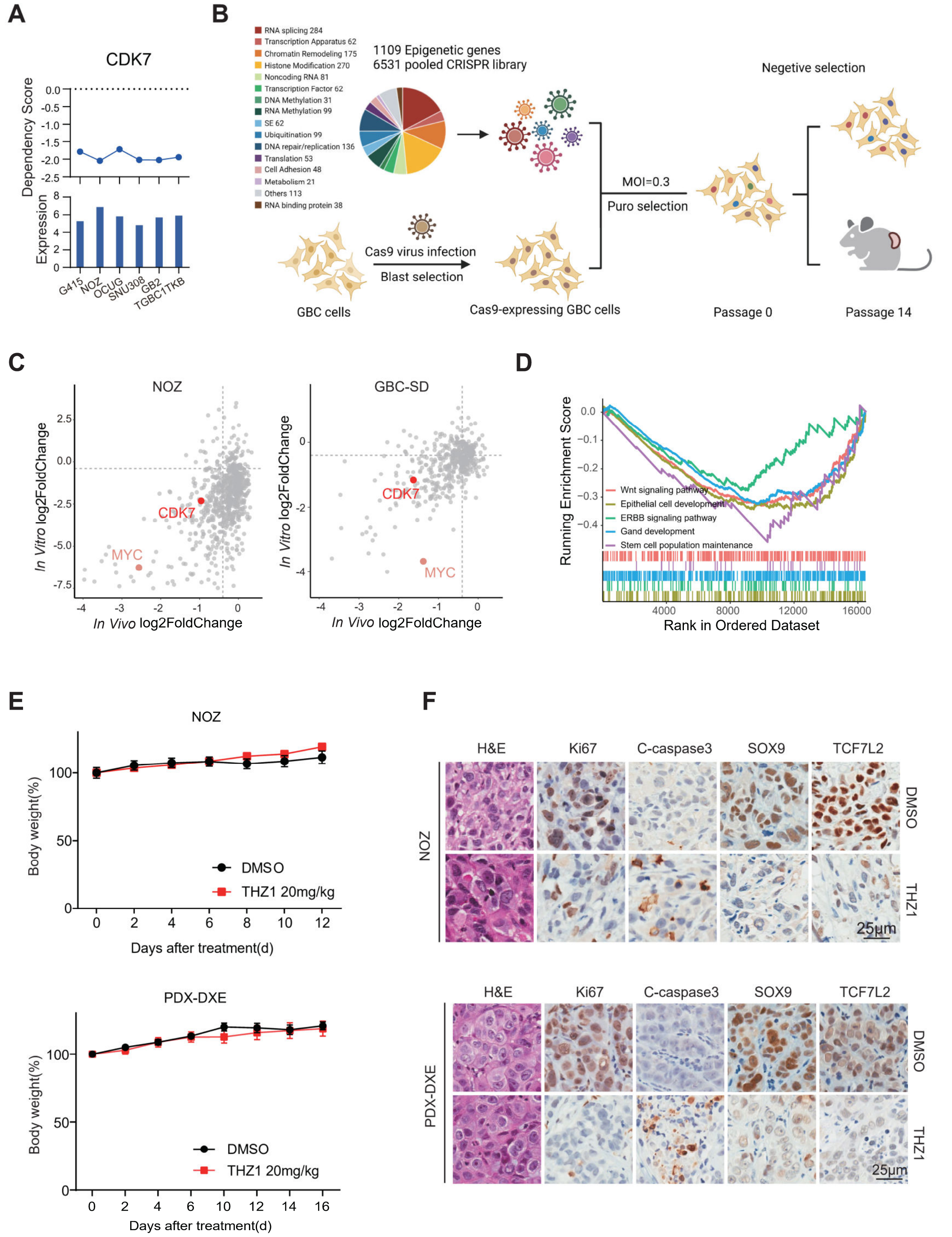

Figure S12 (related to figure 8).

A. The dependency score and expression of CDK7 in 6 GBC cell lines collected in DepMap database.
B. Strategy of CRISPR-Cas9 screening of epigenetic genes library in GBC cell lines.
C. Pooled CRISPR–Cas9 loss-of-function screen results of NOZ and GBC-SD cell line in vitro and in vivo.
D. GSEA results of the top enriched genesets in THZ1 treated NOZ cells versus DMSO treatment.

E. Body weight in mice engrafted with NOZ cells or PDX model with DMSO or THZ1 treatment. Data represent the mean body weight ± SD of (DMSO-treated) or (THZ1-treated) mouse tumors at the indicated time points.

F. Representative images of hematoxylin and eosin (H&E) and immunohistochemical staining for Ki67, cleaved caspase-3, SOX9 or TCF7L2 in tumor paraffin sections.

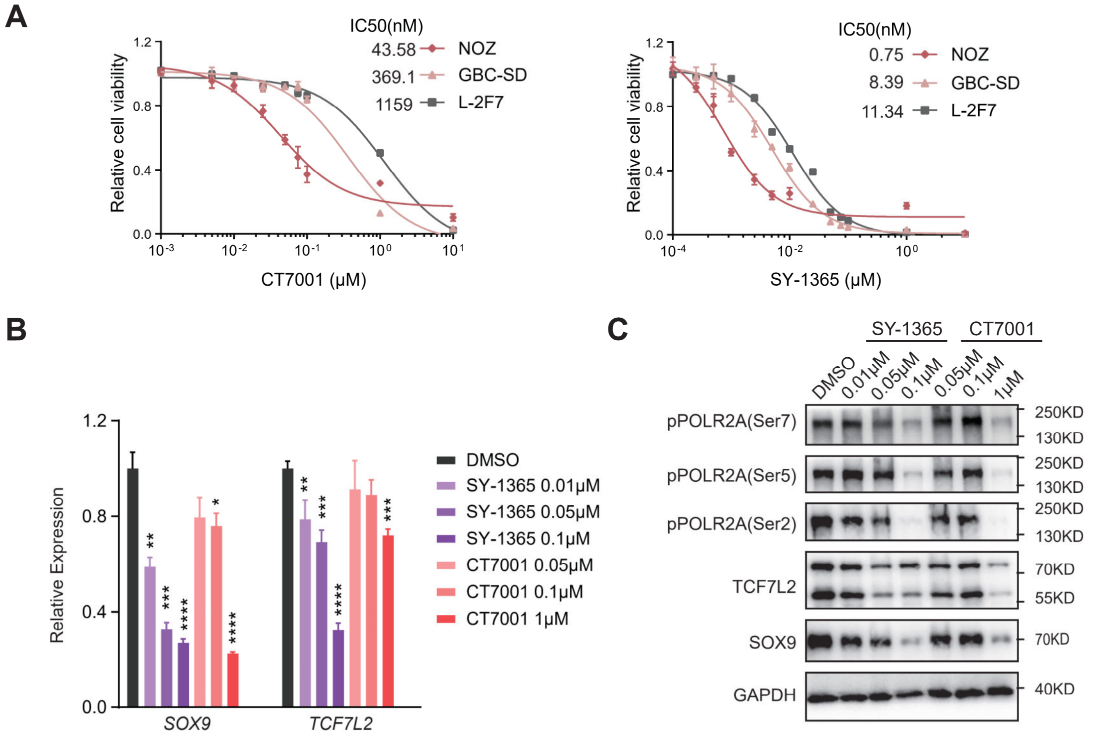

Figure S13 (related to figure 8).

A. Cell viability assay testing CT7001, SY-1365 in GBC and non-malignant cell lines.

B. The qPCR results of *SOX9* and *TCF7L2* expression in NOZ cells treated with DMSO, SY-1365, or CT7001. One-way ANOVA was used for statistical analysis with Dunnett multiple hypothesis test for *p* value correction.

C. Immunoblot analysis of NOZ cells treated with indicated concentrations of DMSO, SY-1365, or CT7001.

Reference

1. Sanjana NE, Shalem O, Zhang F. Improved vectors and genome-wide libraries for CRISPR screening. Nat Methods 2014;11:783-784.
2. Kaya-Okur HS, Wu SJ, Codomo CA, et al. CUT&Tag for efficient epigenomic profiling of small samples and single cells. Nat Commun 2019;10:1930.
3. Corces MR, Trevino AE, Hamilton EG, et al. An improved ATAC-seq protocol reduces background and enables interrogation of frozen tissues. Nat Methods 2017;14:959-962.
4. Schneider CA, Rasband WS, Eliceiri KW. NIH Image to ImageJ: 25 years of image analysis. Nat Methods 2012;9:671-5.
5. Joung J, Konermann S, Gootenberg JS, et al. Genome-scale CRISPR-Cas9 knockout and transcriptional activation screening. Nat Protoc 2017;12:828-863.
6. Li W, Xu H, Xiao T, et al. MAGeCK enables robust identification of essential genes from genome-scale CRISPR/Cas9 knockout screens. Genome Biol 2014;15:554.
7. Wang B, Wang M, Zhang W, et al. Integrative analysis of pooled CRISPR genetic screens using MAGeCKFlute. Nat Protoc 2019;14:756-780.
8. Krueger F, James, F., Ewels, P., Afyounian, E. & Schuster-Boeckler, B. FelixKrueger/TrimGalore: v0.6.7 - DOI via Zenodo (0.6.7). Zenodo., 2021.
9. Kim D, Paggi JM, Park C, et al. Graph-based genome alignment and genotyping with HISAT2 and HISAT-genotype. Nat Biotechnol 2019;37:907-915.
10. Anders S, Pyl PT, Huber W. HTSeq--a Python framework to work with high-throughput sequencing data. Bioinformatics 2015;31:166-9.
11. Love MI, Huber W, Anders S. Moderated estimation of fold change and dispersion for RNA-seq data with DESeq2. Genome Biol 2014;15:550.
12. Yu G, Wang LG, Han Y, et al. clusterProfiler: an R package for comparing biological themes among gene clusters. OMICS 2012;16:284-7.
13. Langmead B, Salzberg SL. Fast gapped-read alignment with Bowtie 2. Nat Methods 2012;9:357-9.
14. Danecek P, Bonfield JK, Liddle J, et al. Twelve years of SAMtools and BCFtools. Gigascience 2021;10.
15. Zhang Y, Liu T, Meyer CA, et al. Model-based analysis of ChIP-Seq (MACS). Genome Biol 2008;9:R137.
16. Ramirez F, Ryan DP, Gruning B, et al. deepTools2: a next generation web server for deep-sequencing data analysis. Nucleic Acids Res 2016;44:W160-5.
17. Thorvaldsdottir H, Robinson JT, Mesirov JP. Integrative Genomics Viewer (IGV): high-performance genomics data visualization and exploration. Brief Bioinform 2013;14:178-92.
18. Loven J, Hoke HA, Lin CY, et al. Selective inhibition of tumor oncogenes by disruption of super-enhancers. Cell 2013;153:320-34.
19. Whyte WA, Orlando DA, Hnisz D, et al. Master transcription factors and mediator establish super-enhancers at key cell identity genes. Cell 2013;153:307-19.
20. Ross-Innes CS, Stark R, Teschendorff AE, et al. Differential oestrogen receptor binding is associated with clinical outcome in breast cancer. Nature 2012;481:389-93.

21. Duttke SH, Chang MW, Heinz S, et al. Identification and dynamic quantification of regulatory elements using total RNA. Genome Res 2019;29:1836-1846.
22. McLean CY, Bristor D, Hiller M, et al. GREAT improves functional interpretation of cis-regulatory regions. Nat Biotechnol 2010;28:495-501.
23. Tanigawa Y, Dyer ES, Bejerano G. WhichTF is functionally important in your open chromatin data? PLoS Comput Biol 2022;18:e1010378.
24. Lin CY, Erkek S, Tong Y, et al. Active medulloblastoma enhancers reveal subgroup-specific cellular origins. Nature 2016;530:57-62.
25. Ott CJ, Federation AJ, Schwartz LS, et al. Enhancer Architecture and Essential Core Regulatory Circuitry of Chronic Lymphocytic Leukemia. Cancer Cell 2018;34:982-995 e7.
26. Quinlan AR, Hall IM. BEDTools: a flexible suite of utilities for comparing genomic features. Bioinformatics 2010;26:841-2.
27. Servant N, Varoquaux N, Lajoie BR, et al. HiC-Pro: an optimized and flexible pipeline for Hi-C data processing. Genome Biol 2015;16:259.
28. Kramer NE, Davis ES, Wenger CD, et al. Plotgardener: cultivating precise multi-panel figures in R. Bioinformatics 2022;38:2042-2045.
29. Ramirez F, Bhardwaj V, Arrigoni L, et al. High-resolution TADs reveal DNA sequences underlying genome organization in flies. Nat Commun 2018;9:189.
30. Wolff J, Rabbani L, Gilsbach R, et al. Galaxy HiCExplorer 3: a web server for reproducible Hi-C, capture Hi-C and single-cell Hi-C data analysis, quality control and visualization. Nucleic Acids Res 2020;48:W177-W184.
31. Wolff J, Bhardwaj V, Nothjunge S, et al. Galaxy HiCExplorer: a web server for reproducible Hi-C data analysis, quality control and visualization. Nucleic Acids Res 2018;46:W11-W16.
32. Salameh TJ, Wang X, Song F, et al. A supervised learning framework for chromatin loop detection in genome-wide contact maps. Nat Commun 2020;11:3428.
33. Zhang Y, Zuo C, Liu L, et al. Single-cell RNA-sequencing atlas reveals an MDK-dependent immunosuppressive environment in ErbB pathway-mutated gallbladder cancer. J Hepatol 2021;75:1128-1141.
34. Stuart T, Butler A, Hoffman P, et al. Comprehensive Integration of Single-Cell Data. Cell 2019;177:1888-1902 e21.
35. Hanzelmann S, Castelo R, Guinney J. GSVA: gene set variation analysis for microarray and RNA-seq data. BMC Bioinformatics 2013;14:7.
36. Garcia-Alonso L, Holland CH, Ibrahim MM, et al. Benchmark and integration of resources for the estimation of human transcription factor activities. Genome Res 2019;29:1363-1375.
37. Gulati GS, Sikandar SS, Wesche DJ, et al. Single-cell transcriptional diversity is a hallmark of developmental potential. Science 2020;367:405-411.
38. Newman AM, Steen CB, Liu CL, et al. Determining cell type abundance and expression from bulk tissues with digital cytometry. Nat Biotechnol 2019;37:773-782.
39. Vivian J, Rao AA, Nothaft FA, et al. Toil enables reproducible, open source, big biomedical data analyses. Nat Biotechnol 2017;35:314-316.
